# The strawberry transcription factor FaRAV1 positively regulates anthocyanin accumulation by activation of *FaMYB10* and anthocyanin pathway genes

**DOI:** 10.1111/pbi.13382

**Published:** 2020-04-13

**Authors:** Zuying Zhang, Yanna Shi, Yuchen Ma, Xiaofang Yang, Xueren Yin, Yuanyuan Zhang, Yuwei Xiao, Wenli Liu, Yunduan Li, Shaojia Li, Xiaofen Liu, Donald Grierson, Andrew C. Allan, Guihua Jiang, Kunsong Chen

**Affiliations:** ^1^ College of Agriculture and Biotechnology Zhejiang University Hangzhou China; ^2^ Zhejiang Provincial Key Laboratory of Horticultural Plant Integrative Biology Zhejiang University Hangzhou China; ^3^ State Agriculture Ministry Laboratory of Horticultural Plant Growth Development and Quality Improvement Zhejiang University Hangzhou China; ^4^ Institute of Horticulture Zhejiang Academy of Agricultural Sciences Hangzhou China; ^5^ College of Mathematical Science Zhejiang University Hangzhou China; ^6^ Division of Plant and Crop Sciences School of Biosciences University of Nottingham Loughborough UK; ^7^ The New Zealand Institute for Plant & Food Research Limited Auckland New Zealand; ^8^ School of Biological Sciences University of Auckland Auckland New Zealand

**Keywords:** RAV, anthocyanin, activator, strawberry, MYB

## Abstract

The RAV (related to ABI3/viviparous 1) group of transcription factors (TFs) play multifaceted roles in plant development and stress responses. Here, we show that strawberry (*Fragaria* × *ananassa*) *FaRAV1* positively regulates anthocyanin accumulation during fruit ripening via a hierarchy of activation processes. Dual‐luciferase assay screening of all fruit‐expressed *AP2/ERFs* showed FaRAV1 had the highest transcriptional activation of the promoter of *FaMYB10*, a key activator of anthocyanin biosynthesis. Yeast one‐hybrid and electrophoretic mobility shift assays indicated that FaRAV1 could directly bind to the promoter of *FaMYB10*. Transient overexpression of *FaRAV1* in strawberry fruit increased *FaMYB10* expression and anthocyanin production significantly. Correspondingly, transient RNA interference‐induced silencing of *FaRAV1* led to decreases in *FaMYB10* expression and anthocyanin content. Transcriptome analysis of *FaRAV1*‐overexpressing strawberry fruit revealed that transcripts of phenylpropanoid and flavonoid biosynthesis pathway genes were up‐regulated. Luciferase assays showed that FaRAV1 could also activate the promoters of strawberry anthocyanin biosynthetic genes directly, revealing a second level of *FaRAV1* action in promoting anthocyanin accumulation. These results show that *FaRAV1* stimulates anthocyanin accumulation in strawberry both by direct activation of anthocyanin pathway gene promoters and by up‐regulation of *FaMYB10*, which also positively regulates these genes.

## Introduction

The octoploid cultivated strawberry (*Fragaria* × *ananassa*) is a typical non‐climacteric fruit and an economically important horticultural crop worldwide. Its popularity is principally due to its sweet taste, unique fragrance, nutritional value and bright colour, all of which are pivotal factors in determining fruit quality.

Strawberry fruit when ripe are distinguished by a high content of anthocyanins, which are water‐soluble flavonoid compounds that generate the characteristic reddish, purple and bluish hues of many fruits, leaves, flowers and seeds. Extensive studies have revealed diverse biological functions for anthocyanins, including an association with reduced incidence of chronic diseases, an ability to confer stress resistance, and attraction of pollinators and seed dispersers (Schaefer *et al* *.*, 2004). Anthocyanins are synthesized from phenylalanine by a series of enzymes: phenylalanine ammonia‐lyase (PAL), cinnamate 4‐hydroxylase (C4H), 4‐coumarate: CoA ligase (4CL). The specific flavonoid pathway is initiated by the condensation of one molecule of 4‐coumaroyl‐coenzyme A (CoA) and three molecules of malonyl‐CoA, catalysed by chalcone synthase (CHS) to produce naringenin chalcone. Other early anthocyanin biosynthetic genes (EBG) include chalcone isomerase (CHI), flavonoid 3‐hydroxylase (F3H/FHT) and flavonoid 3′‐hydroxylase (F3′H), which produce naringenin, dihydrokaempferol and dihydroquercetin, respectively. The late anthocyanin biosynthetic pathway genes (LBG) include dihydroflavonol‐4‐reductase (DFR), anthocyanidin synthase (ANS) and 3‐glycosyltransferase (GT1) to produce leucoanthocyanidins, anthocyanidins and anthocyanins, respectively (Almeida *et al* *.*, 2007; Griesser *et al.*, 2008; Lin *et al* *.*, 2013). The activity of the MYB‐bHLH‐WD40 (MBW) ternary transcriptional complex is central to the regulation of the pathway. This consists of three classes of regulatory proteins, R2R3‐MYBs, bHLHs and TTG1 (also termed WD40), which can act independently or in cooperation with each other as a complex (Baudry *et al* *.*, 2004; Lloyd *et al* *.*, 2017; Xu *et al* *.*, 2015). MYB transcription factors are the most important regulators of anthocyanin biosynthesis (Allan *et al* *.*, 2008). In maize, the R2R3 MYB C1 protein interacts with a bHLH TF to activate the promoter of *DFR* (Sainz *et al* *.*, 1997). In *Arabidopsis*, anthocyanin production is transcriptionally regulated by an R2R3‐MYB protein, for example PAP1, PAP2, MYB113 or MYB114; one bHLH protein, for example TT8, GL3 or EGL3; and one TTG1 protein (Gonzalez *et al* *.*, 2008; Li, 2014). In apple, *MdMYB1*/*MYBA* TF transcripts are correlated with apple fruit skin colour and *MdMYB10* is responsible for production of anthocyanin in red‐fleshed fruit (Ban *et al.*, 2007; Espley *et al.*, 2007; Takos *et al.*, 2006). In grapevine, VvMYB1 and VvMYB*2* act mainly on the expression of *VvUFGT* to regulate anthocyanin biosynthesis (Kobayashi *et al.*, 2002, 2004; Walker *et al.*, 2007). In pear, PpMYB10 enhances anthocyanin accumulation via regulation of genes encoding enzymes of the anthocyanin pathway (Feng *et al.*, 2010). In tomato, *LeANT1* (Sapir *et al.*, 2008) and *LeAN2* (Boches *et al.*, 2009; Mes *et al.*, 2008) can also regulate anthocyanin biosynthesis. Many other anthocyanin‐regulating MYBs have been isolated from other species, for example *Petunia hybrida AN2* (Quattrocchio *et al.*, 1999), *Gerbera hybrida GhMYB10* (Elomaa *et al.*, 2003), *Oryza sativa C1* (Saitoh *et al.*, 2004), *Antirrhinum majus ROSEA1*, *ROSEA2* and *VENOSA* (Schwinn *et al.*, 2006) and *Garcinia mangostana GmMYB10* (Palapol *et al.*, 2009).

In strawberry, several transcription factors have been identified and functionally confirmed as regulators of anthocyanin biosynthesis. FaMYB1 and FcMYB1 (from *Fragaria chiloensis*) both act as repressors of genes that catalyse the few steps of anthocyanin biosynthesis in strawberry (Aharoni *et al.*, 2001; Salvatierra *et al.*, 2013). Diploid (*Fragaria vesca*) transgenic strawberry plants in which *FvMYB10* was inhibited had undetectable levels of anthocyanin, while overexpression of *FvMYB10* significantly elevated anthocyanin levels, confirming the crucial role of *MYB10* in regulating anthocyanin accumulation (Lin‐Wang *et al.*, 2014). High‐throughput transcriptome analysis showed that *FaMYB10* is a general regulator of EBG and LBG in the flavonoid/phenylpropanoid pathway during the ripening of strawberry (Medina‐Puche *et al.*, 2014). Moreover, FvMYB10 interacts with FvbHLH33 to activate the *FvDFR* and *FvUFGT* promoter (Lin‐Wang *et al.*, 2014). Through systematic analysis of SNP variants, a candidate SNP in *FvMYB10* was confirmed to be the cause of the loss of red colour in yellow strawberry fruits (Hawkins *et al.*, 2016). An ACTTATAC insertion introduces a predicted premature termination codon in *FaMYB10*, which suggested the loss of FaMYB10 intact protein accounts for the loss of red colour in white octoploid strawberry (Wang *et al.*, 2019a). Thus, all the evidence indicates that *MYB10* plays a key role in strawberry anthocyanin biosynthesis.

AP2/ERFs are plant‐specific transcription factors with diverse functions in plant growth, development and responses to environmental stresses. They are divided into four categories: the APETALA2 (AP2), ERF, related to ABI3/VP1 (RAV), and Soloist families (Licausi *et al.*, 2013; Mizoi *et al.*, 2012; Zhu *et al.*, 2010). AP2/ERFs have been implicated in determining different aspects of fruit quality, including fruit aroma and flavour (Xie *et al.*, 2016). Our previous research characterized citrus *CitAP2.10* as a regulator of (+)‐valencene synthesis (Shen *et al.*, 2016) while citrus CitERF13 interacts with CitVHA‐c4 to regulate citric acid accumulation (Li *et al.*, 2016). Several AP2/ERFs have been shown to be involved in anthocyanin biosynthesis in different species. In pear, PpERF3 interacts with PpMYB114 and PpbHLH3 to co‐regulate the coloration of red pear fruit (Yao *et al.*, 2017) and Pp4ERF24 and Pp12ERF96 regulate blue light‐induced anthocyanin biosynthesis in ‘Red Zaosu’ pear fruit by interacting with PpMYB114, thus enhancing the expression of *PpUFGT* (Ni *et al* *.*, 2019). In apple, the regulator MdERF1B not only interacts with MdMYB9 and MdMYB11 but also binds to the promoters of *MdMYB9* and *MdMYB11* to promote anthocyanin and proanthocyanin accumulation (Zhang *et al.*, 2018a). Another ERF transcription factor MdERF38 promotes drought stress‐induced anthocyanin biosynthesis via interaction with MdMYB1 and can be degraded by MdBT2 at the post‐translational level (An *et al.*, 2019). Several AP2/ERFs have been identified as having roles in determining strawberry fruit quality. FaABI4 is an AP2‐type protein and a positive regulator of strawberry ripening (Chai and Shen, 2016), and an ERF–MYB complex including FaERF9 and FaMYB98 activates the *FaQR* promoter and increases furaneol content in cultivated strawberry (Zhang *et al.*, 2018b). However, it is still unknown whether *FaAP2/ERFs* participate in anthocyanin biosynthesis in strawberry.

In this study, we screened all *AP2/ERF* genes expressed in strawberry fruit by dual‐luciferase assay to identify those capable of activating the promoter of *FaMYB10* and found FaRAV1 had the highest activation effect. FaRAV1 was found to directly bind to the *FaMYB10* promoter. Transient overexpression and RNA interference (RNAi) of *FaRAV1* in strawberry fruit validated its significant role in anthocyanin biosynthesis. In addition, *FaRAV1* also directly transactivates the promoters of anthocyanin biosynthesis‐related genes, which illustrates the importance of *FaRAV1* in strawberry anthocyanin biosynthesis.

## Results

### Regulatory effect of FaAP2/ERFs on the promoter of *FaMYB10*

**Figure 1:**
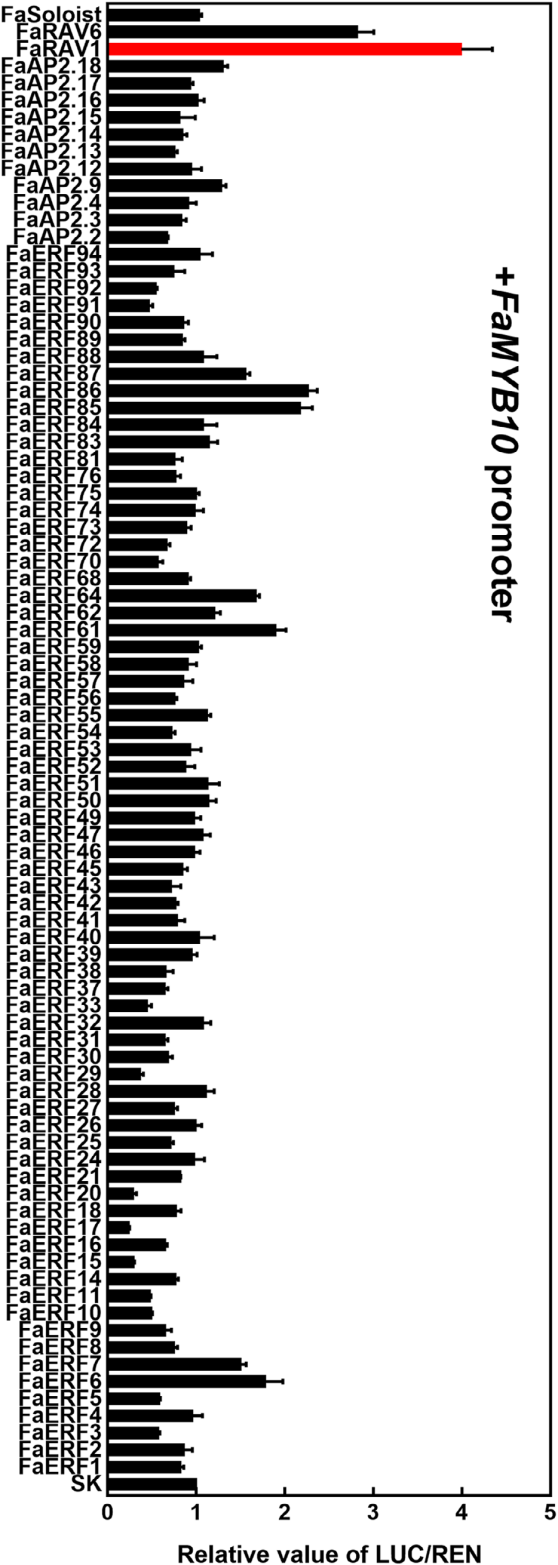
Regulatory effects of *FaAP2/ERFs* on the promoter of *FaMYB10.* SK refers to empty vector and is set as 1. Error bars represent SE based on three biological replicates.

In a previous study, 120 individual FaAP2/ERF genes from ‘Yuexin’ strawberry were isolated and identified, consisting of 95 ERFs, 18 AP2s, 6 RAVs and 1 soloist member (Zhang *et al.*, 2018b). It was found that 86 of these *AP2/* *ERFs* were expressed in strawberry fruit. Using this information, we screened all the fruit‐expressed *AP2/ERFs* for their ability to transactivate the promoter of *FaMYB10.* The results (threshold was set as 2) showed that 5 members could activate the promoter of *FaMYB10*, including FaRAV1, FaRAV6, FaERF61, FaERF85 and FaERF86 (Figure 1), with FaRAV1 displaying the strongest activation effect of approximately 4.0‐fold. According to a phylogenetic tree containing strawberry AP2/ERFs and other ERFs which had been characterized in anthocyanin biosynthesis, FaERF85 and FaERF86 were similar to MdERF1B (Figure S1).

The strawberry RAV family has six members. A phylogenetic tree of strawberry RAV genes was constructed by aligning the full‐length amino acid sequences with other plant RAVs, including Arabidopsis, Populus, rice, apple and tomato (Figure S2). FaRAV1 clustered with FaRAV2 and FaRAV3 and was similar to SlRAV3, although none of these genes have been characterized to date. Transient expression in tobacco leaves of 35S‐FaRAV1‐GFP showed strong fluorescence in the nucleus, and the red nucleus signal of the mCherry marker merged with the green fluorescence (Figure S3).

### The interaction between FaRAV1 and the *FaMYB10* promoter

**Figure 2:**
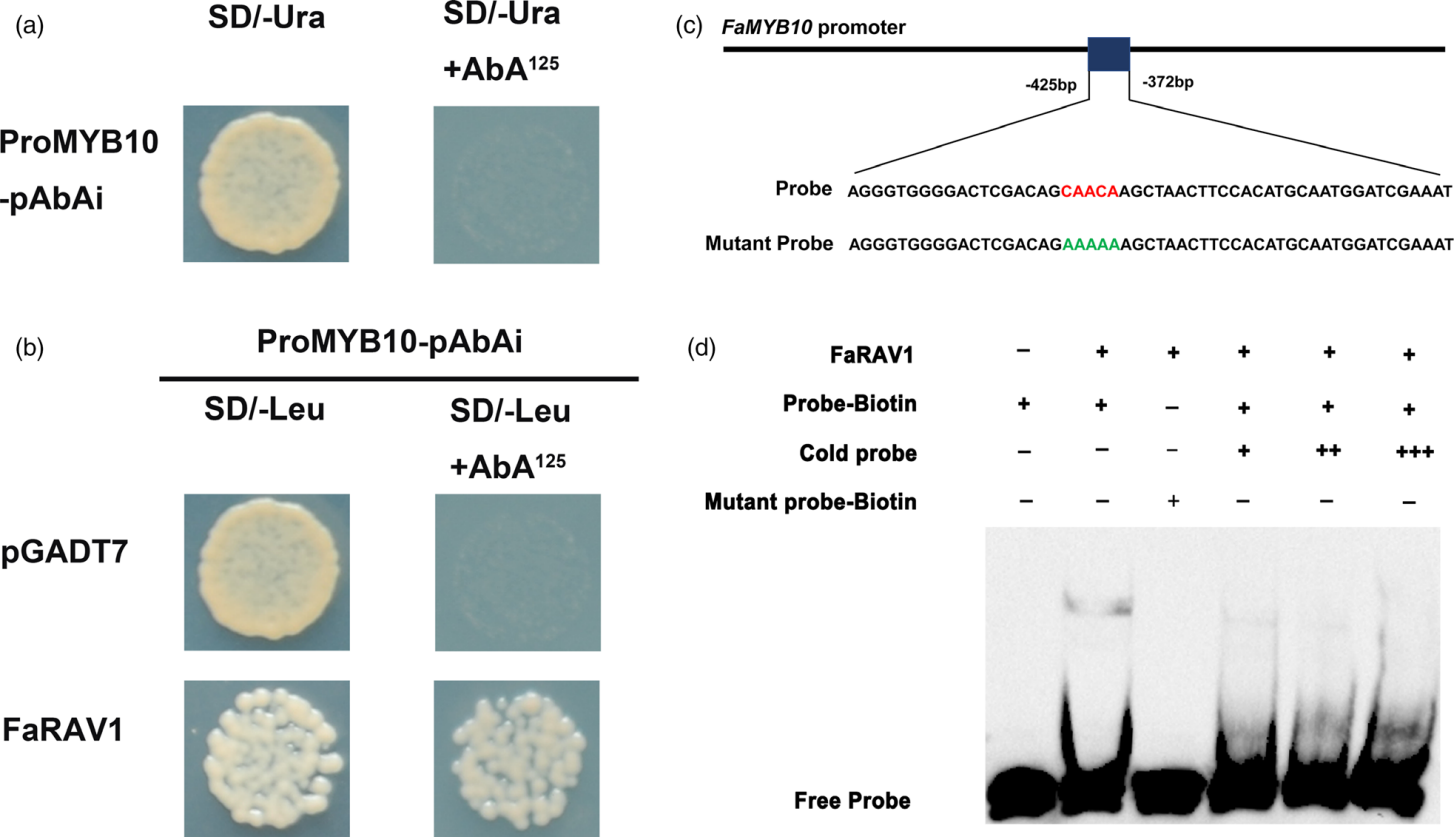
The interaction between FaRAV1 and the *FaMYB10* promoter. a, Autoactivation was tested on SD/‐Ura in the presence of 125 ng/mL aureobasidin A (AbA). b, Physical interaction was determined on SD medium lacking Leu in the presence of 125 ng/mL AbA. The empty pGADT7 vector was applied as a negative control. c, The probe used for EMSA with the RAV core sequence is in red, and the mutated nucleotides indicated in green. D, EMSA of 3′‐biotin‐labelled dsDNA probes with the purified FaRAV1 protein. The presence (+) or absence (–) of specific probes is marked. The concentration of the cold probe was 16 nm (+), 32 nm (++) or 64 nm (+++) while that of the biotinylated probe was 8 nm. Water was added in place of FaRAV1 protein as a control.

According to the results of the dual‐luciferase assay, FaRAV1, which showed the strongest activation effect on the *FaMYB10* promoter, was chosen for further study. The promoter sequence of *FaMYB10* was inserted into the pAbAi vector (Clontech, Japan) and transformed into the Y1H strain. The *FaMYB10* promoter was not activated without protein binding (Figure 2a). The interaction between FaRAV1 and the *FaMYB10* promoter was analysed; yeast containing FaRAV1 exhibited normal growth under 125 ng/mL AbA, while growth of the negative control cells, containing the empty vector, was inhibited, which indicated that FaRAV1 could directly bind to the promoter of *FaMYB10* (Figure 2b). The specificity of FaRAV1 binding to the *FaMYB10* promoter was confirmed by electrophoretic mobility shift assay (EMSA). It has been reported that RAV genes contain an AP2 domain and a B3 domain, which can bind specifically to DNA sequences with the consensus motif, 5′‐CAACA‐3′ and 5′‐CACCTG‐3′ respectively (Kagaya *et al.*, 1999). After searching the promoter of *FaMYB10*, we found one CAACA motif within a 402‐bp region upstream of the start codon (Figure 2c). EMSA results indicated that FaRAV1 can bind to this specific CAACA motif and mutating the putative binding sites eliminated FaRAV1 protein binding, while increasing the concentration of cold probe significantly reduced the binding affinity of the biotinylated probe (Figure 2d).

### Relative expression of *FaRAV1*,* FaMYB10* and total anthocyanin content, during strawberry fruit development and ripening

**Figure 3:**
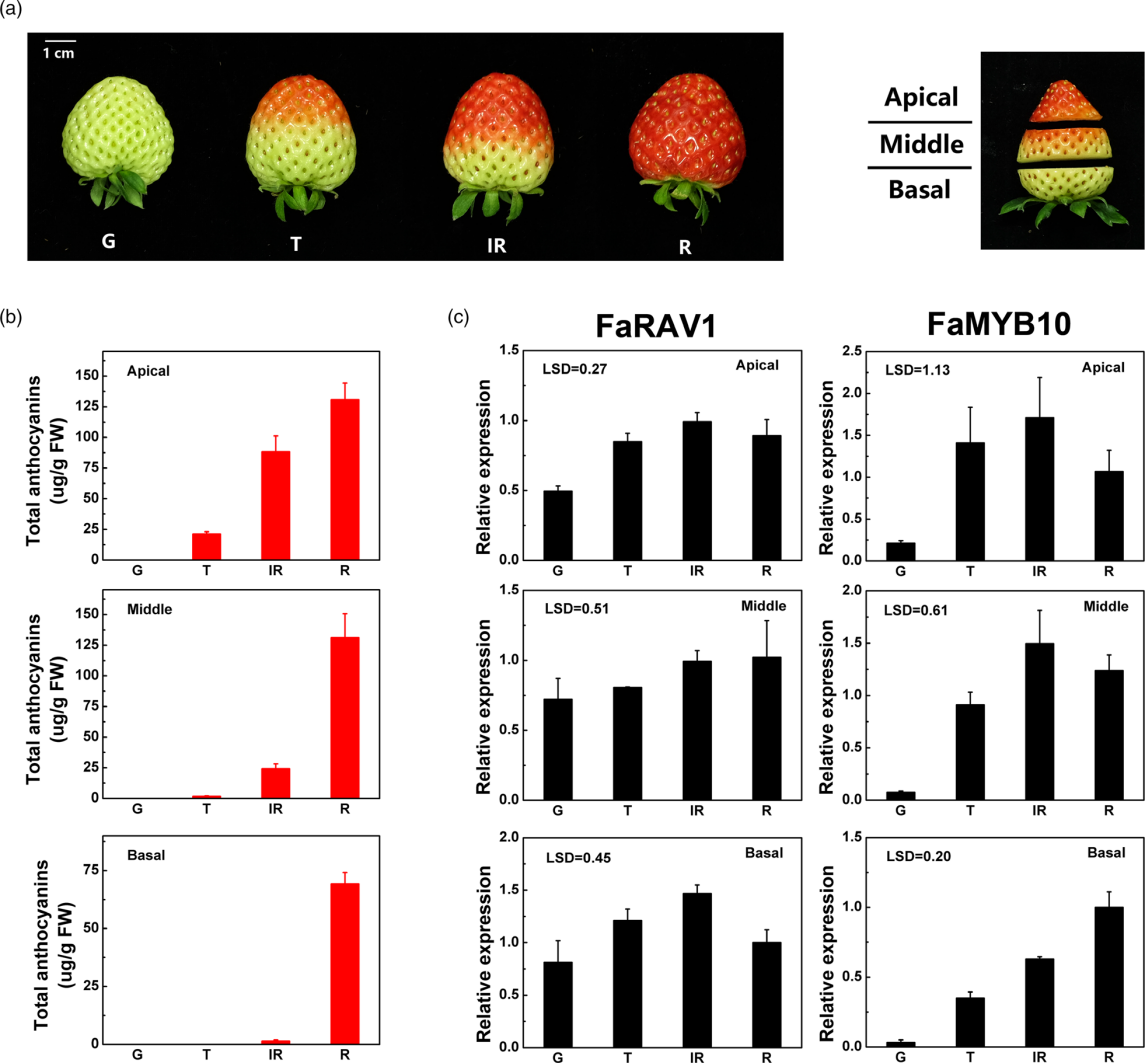
Relative expression of *FaRAV1*, *FaMYB10* and total anthocyanins during strawberry (cv. Yuexin) fruit development and ripening. a, Fruits were collected at four stages: G, green; T, turning; IR, intermediate red; and R, full red. Three sections were sampled: apical; middle; and basal. b, Changes in the contents of total anthocyanins in each of the three sections at four developmental stages. FW, fresh weight. C, Relative expression levels of *FaRAV1* and *FaMYB10*. The expression levels were calculated relative to corresponding values in the basal section at the R stage. Error bars represent SE based on three biological replicates. LSD values represent LSD at *P* = 0.05.

The process of strawberry fruit development and ripening were divided into four major stages: G (green), T (turning), IR (intermediate red) and R (full red). Fruit from each stage were cut into three parts (apical, middle and basal) for further analysis (Figure 3a). Anthocyanin accumulation was initiated in the apical sections. At the turning stage, anthocyanin accumulated mainly in the apical region (21.3 µg/g), less than 10% (1.76 µg/g) in the middle section and no detectable anthocyanin in the basal region (Figure 3b). At the ripening stage, the anthocyanin content was 130.8, 131.1 and 69.3 µg/g in the apical, middle and basal sections, respectively. The transcript of *FaRAV1* increased during colour change in the apical, middle and basal sections and increased steadily during fruit ripening. The transcript of *FaMYB10*, in contrast, showed an expression pattern which preceded the accumulation of anthocyanin (Figure 3c).

### Transient overexpression of *FaRAV1* promotes anthocyanin accumulation

**Figure 4:**
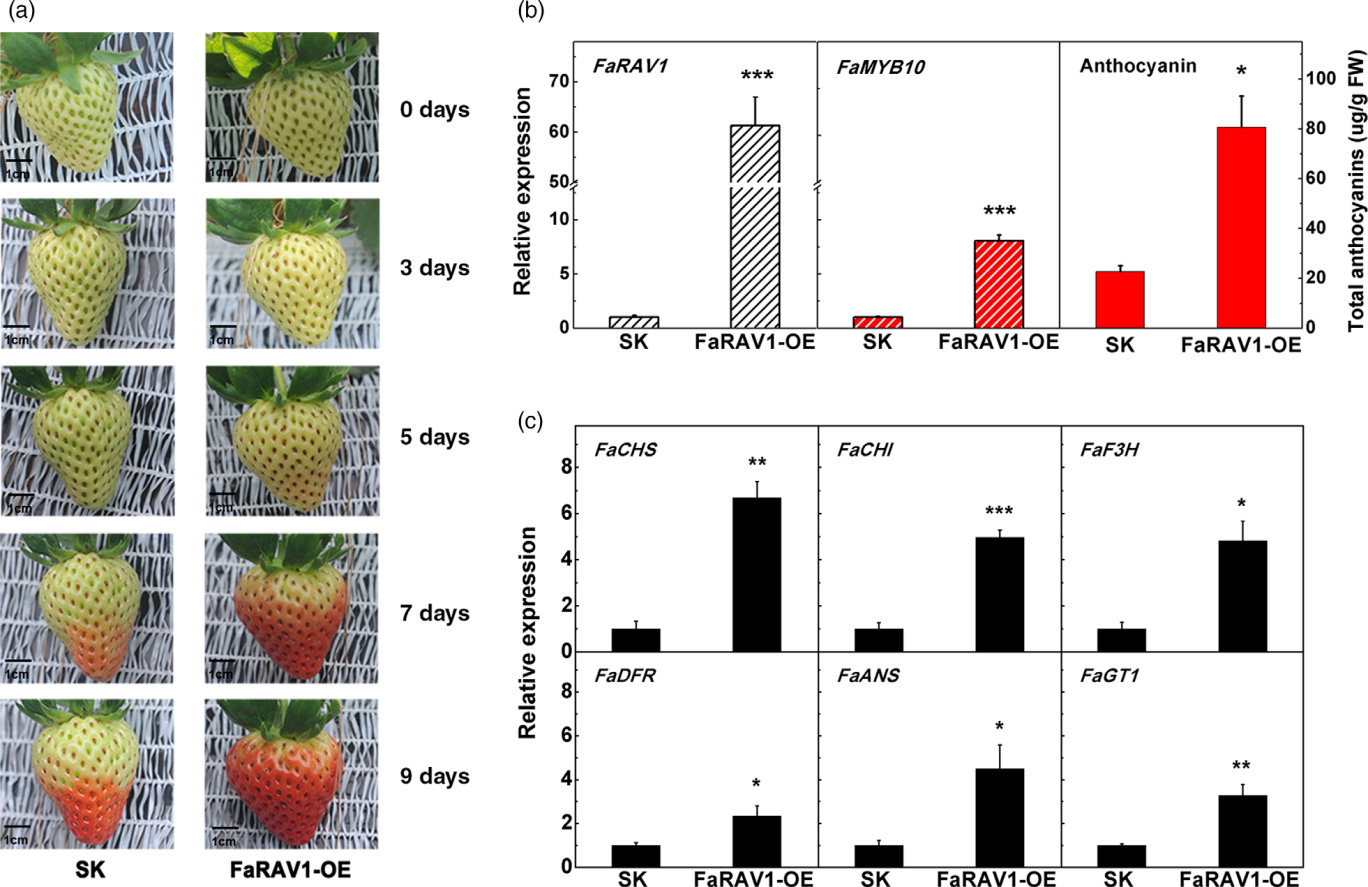
Transient overexpression of *FaRAV1* promotes anthocyanin accumulation in strawberry fruit. a, Transient overexpression of *FaRAV1* (right) and control (left) fruit at different times after injection, SK refers to empty vector. b, Transcript analysis of *FaRAV1*, *FaMYB10* and anthocyanin quantification of *FaRAV1* OE fruit and control fruit. C, RT‐qPCR verification of transcript levels of the main anthocyanin‐related genes. Error bars represent SE based on three biological replicates. Asterisks denote significant differences using Student’s *t*‐test, *P < 0.05, **P < 0.01, ***P < 0.001.

To test the relationship between *FaRAV1* and anthocyanin accumulation, we transiently expressed *FaRAV1* by injection of *Agrobacterium tumefaciens* containing the *FaRAV1* OE construct driven by the 35S promoter into attached green strawberry fruit. Overexpression of *FaRAV1* significantly promoted anthocyanin accumulation (Figure 4a); 5 days after infiltration *FaRAV1* OE fruit started to turn red at the apical end and became almost fully red after 7 days, whereas the control fruit was just beginning to turn red. On the ninth day, *FaRAV1* OE fruit became totally red, while the control fruit reached approximately intermediate red stage. The fruit were harvested 9 days after infiltration (Figure S4) and the relative expression of *FaRAV1* was increased in OE fruit, up to 60‐fold compared to the control, indicating the transient overexpression was very effective (Figure 4b). *FaMYB10*, which has been shown to be a direct target of *FaRAV1* (Figures 1 and 2) was induced 8.1‐fold (Figure 4b). The total anthocyanin content of *FaRAV1* OE fruit was up to 3.6‐fold higher compared with the control fruit (Figure 4b), supporting a role for *FaRAV1* in promoting anthocyanin accumulation. KEGG pathway analysis of the differentially expressed genes (DEGs) between *FaRAV1* OE and control fruit revealed enrichment of genes involved in phenylpropanoid biosynthesis and flavonoid biosynthesis using octoploid cultivated strawberry as reference genome (Edger *et al.*, 2019) (Figure S5), which is consistent to results using diploid strawberry as reference genome (data not shown). Thus, we checked the expression levels of genes encoding enzymes of the anthocyanin pathway by RT‐qPCR and found *CHS*, *CHI*, *F3H*, *DFR*, *ANS* and *GT1* showed 6.7‐, 5.0‐, 4.8‐, 2.4‐, 4.5‐ and 3.3‐fold higher levels of expression respectively (Figure 4c), which was consistent with the RNA‐seq data.

### Transient RNAi of *FaRAV1* down‐regulates anthocyanin biosynthesis

**Figure 5:**
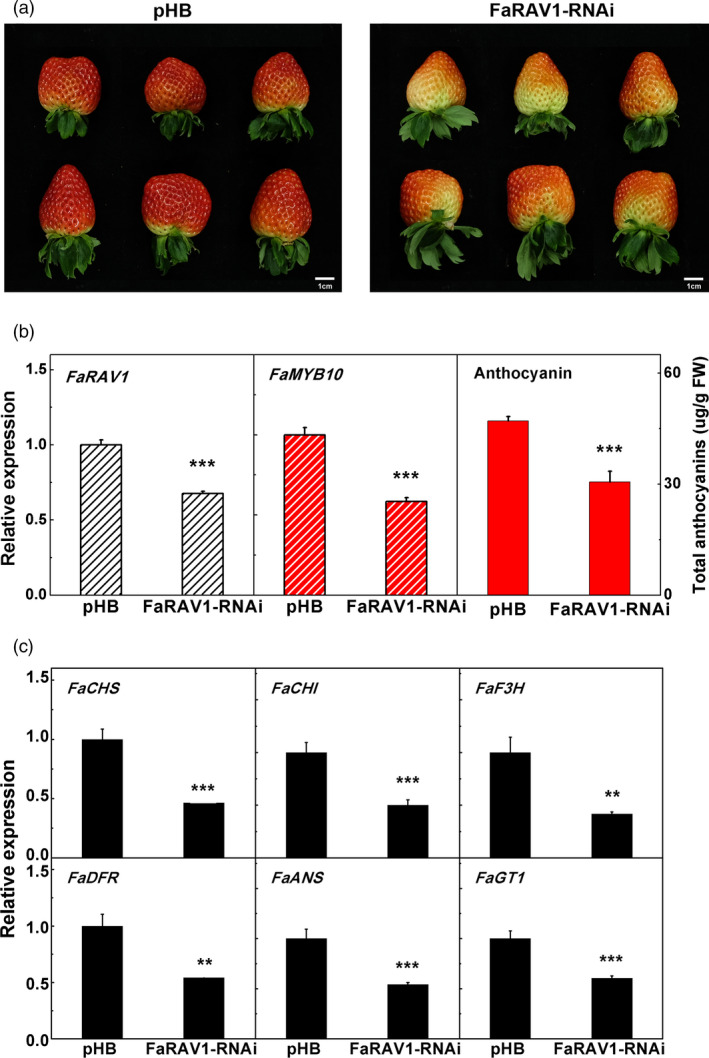
Transient RNAi of *FaRAV1* reduced anthocyanin contents in strawberry fruit. a, The phonotype of transient RNAi of *FaRAV1* (right) and control (left) fruit. b, Transcript analysis of *FaRAV1*, *FaMYB10* and anthocyanin quantification of *FaRAV1* RNAi fruit and control fruit. c, RT‐qPCR verification of transcript levels of the main anthocyanin‐related genes. Error bars represent SE based on three biological replicates. Asterisks denote significant differences using Student’s *t*‐test, **P < 0.01, ***P < 0.001.

For a better understanding of the function of *FaRAV1*, we performed transient RNAi (Wang *et al*., 2019b) . *Agrobacterium tumefaciens* strain GV3101 harbouring *FaRAV1* RNAi construct and empty vector pHB were separately injected into attached green strawberry fruit. RNAi fruit showed less coloration, and the content of anthocyanin was reduced to 65% compared to the control (Figure 5a,b). The expression level of *FaRAV1* was reduced to 68% (Figure 5b). Transcripts of *FaMYB10*, the direct target gene of *FaRAV1*, were decreased significantly (Figure 5b). RT‐qPCR analysis showed that transcripts of all selected anthocyanin biosynthesis‐related genes were also decreased in *FaRAV1* RNAi fruit (Figure 5c). Consistent with the transient overexpression of *FaRAV1*, RNAi results demonstrated that *FaRAV1* plays an important role in the regulation of strawberry anthocyanin biosynthesis.

### Regulatory role of *FaRAV1* in activating anthocyanin biosynthetic gene promoters

**Figure 6:**
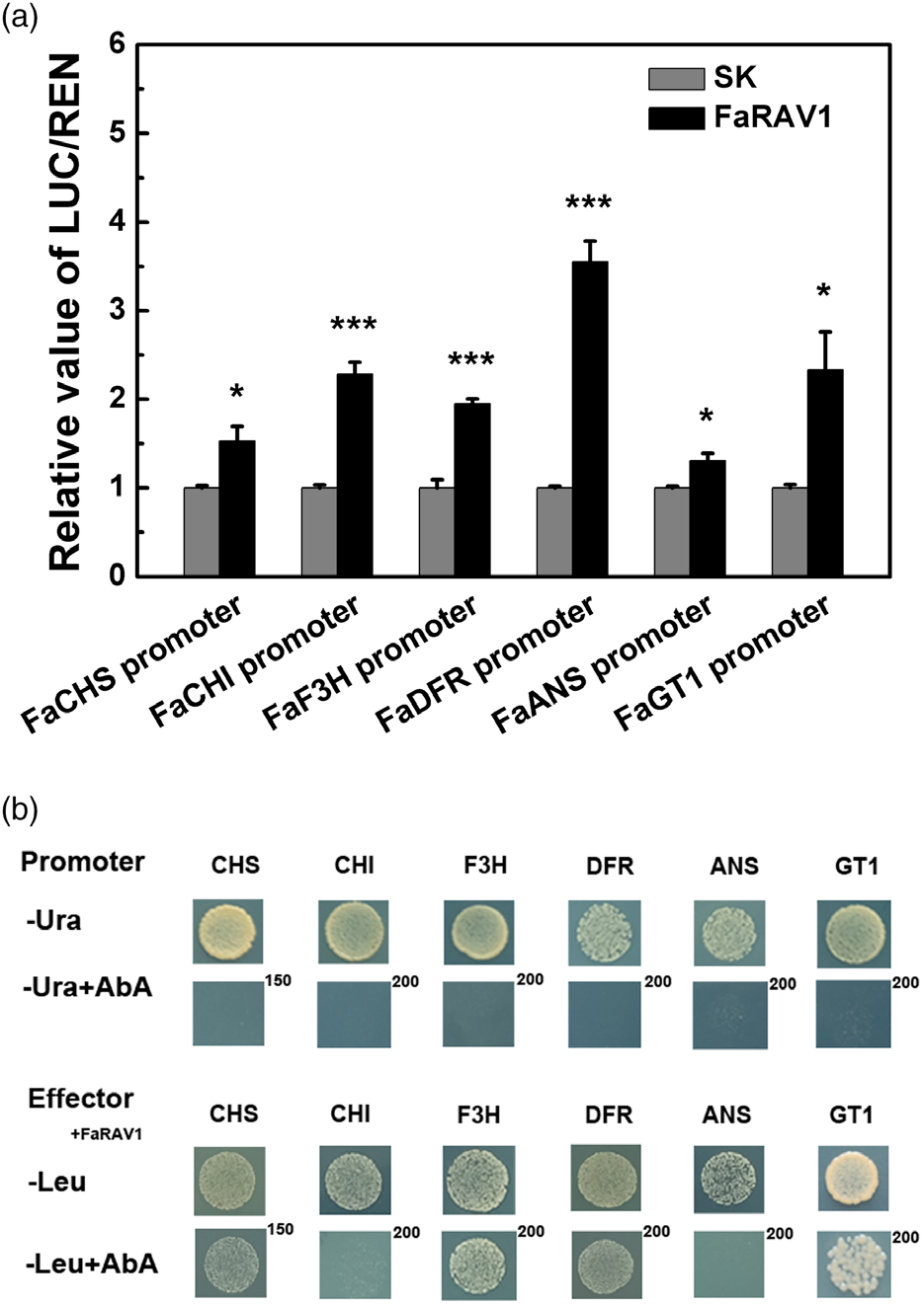
Effect of FaRAV1 on the promoters of anthocyanin biosynthetic genes. a, Dual‐luciferase assay of FaRAV1 on the promoters of anthocyanin biosynthetic genes. Error bars represent SE based on three biological replications. Asterisks denote significant differences using Student’s *t*‐test, *P < 0.05, ***P < 0.001. b, Yeast one‐hybrid analysis of the interaction of FaRAV1 and anthocyanin biosynthetic gene promoters. All promoters were used for autoactivation tests in the presence of different concentrations of aureobasidin A (AbA) on SD/‐Ura medium, and physical interaction was determined on SD/‐Leu medium in the presence of corresponding AbA concentrations.

To further explore the trans‐regulatory role of *FaRAV1* in anthocyanin biosynthesis, we assayed the transactivation effect of *FaRAV1* on the promoters of anthocyanin biosynthetic genes. Dual‐luciferase assays revealed that FaRAV1 could significantly activate the promoters of *CHS* (1.53‐fold), *CHI* (2.3‐fold), *F3H* (1.95‐fold)*, DFR* (3.6‐fold), *ANS* (1.31‐fold) and *GT1* (2.3‐fold)*,* respectively and that FaRAV1 showed a higher activation effect towards the *DFR* promoter compared with the other promoters (Figure 6a). Yeast one‐hybrid assay showed that FaRAV1 directly bind to the *CHS*, *F3H*, *DFR* and *GT1* promoters but not promoters of *CHI* and *ANS* (Figure 6b), indicating that *FaRAV1* would promote anthocyanin accumulation by direct regulation of *CHS*, *F3H*, *DFR*, *GT1* and indirect regulation of *CHI*, *ANS*, in addition to regulating *FaMYB10* transcript accumulation.

## Discussion

### FaRAV1 positively regulates the biosynthesis of anthocyanin by direct activation of *FaMYB10* transcription

Previous studies have shown that accumulation of RAV transcripts can be induced by darkness, wounding, low temperature, drought stress and pathogen attack (Fowler *et al.*, 2005; Lee *et al.*, 2005; Li *et al.*, 2011; Sohn *et al.*, 2006). Moreover, RAVs have been implicated in different aspects of plant physiological and developmental responses. *SlRAV2,* for example, increases bacterial wilt tolerance by inducing the expression of PR genes in tomato (Li *et al.*, 2011). *RAV1* can also regulate hypocotyl elongation (Ma *et al.*, 2005) and has been suggested to inhibit plant growth (Hu *et al.*, 2004), regulate leaf senescence (Woo *et al.*, 2010) and control bud outgrowth in poplar (Moreno‐Cortés *et al.*, 2012). TEMPRANILLO genes (*TEM1* and *TEM2*) repress floral induction in the photoperiod pathway in *Arabidopsis* (Castillejo and Pelaz, 2008).

RAVs have not been implicated in fruit anthocyanin production or other aspects of fruit quality. In apple fruit, *MdRAV1* is regulated by MdWRKY31 and is involved in mediating abscisic acid (ABA) sensitivity (Zhao *et al.*, 2019). Anthocyanin can be induced by some abiotic stresses, such as drought (Hughes *et al.*, 2010), ABA (Jia *et al.*, 2011), light (Azuma *et al.*, 2012), cold temperature (Steyn *et al.*, 2009) and wounding stress (Gan *et al.*, 2014; Saltveit, 2000). *Cis*‐elements related to abiotic stress responses are present in the promoter of *FaRAV1*, such as those implicated in response to ABA, methyl jasmonic acid (MeJA) and light response, with *cis*‐elements involved in light responsiveness appearing most frequently, providing clues for investigating the role of *FaRAV1* (Table S3). Previous studies have reported that ABA is an internal signal for strawberry fruit ripening (Jia *et al.*, 2011), and RAV transcription factors also play an important role in stress responses. Thus, we examined the expression of *FaRAV1* in response to ABA in fruits. As is shown in Figure S6, *FaRAV1* expression was sensitive to ABA, and transcripts were induced by 50 μm (6 h treatment) and 100 μm ABA (1 or 6 h treatment), indicting FaRAV1 might be involved in ABA‐mediated coloration and the mechanism of how *FaRAV1* regulates needs further study. Here, we showed that FaRAV1 participates in anthocyanin accumulation via direct binding to the promoter of *FaMYB10* and had the strongest activation effect on the *FaMYB10* promoter compared with other fruit‐expressed *FaAP2/ERFs* (Figures 1 and 2). In addition, expression of *FaMYB10* was induced in *FaRAV1* OE fruit and reduced in *FaRAV1* RNAi fruit correspondingly, indicating that *FaRAV1* positively regulates anthocyanin biosynthesis via *FaMYB10*.

### Other potential *FaAP2/ERFs* regulators of anthocyanin production in strawberry

By dual‐luciferase assay (Figure 1), we found FaRAV6, FaERF61, FaERF85, FaERF86 could also activate the *FaMYB10* promoter (with a cut‐off of two‐fold). There were also four *FaAP2/ERFs* that appear to repress transcriptional activity of the *FaMYB10* promoter: FaERF15, FaERF17, FaERF20 and FaERF29 (threshold set as 0.4). Based on transcript analysis of fruit‐expressed *FaAP2/ERFs* in a previous paper (Zhang *et al* *.*, 2018b), it could be suggested that *FaERF85* might be a positive anthocyanin regulator, as its transcript greatly increased at the turning stage of strawberry fruit ripening. Based on phylogenetic analysis, FaERF85 clustered with MdERF1B, a positive anthocyanin regulator in apple (Zhang *et al.*, 2018a), further supporting the suggestion of the involvement of FaERF85 in anthocyanin accumulation. Transcripts of *FaERF29* gradually decreased during fruit development, which is negatively related to anthocyanin content, implying it might be a repressor of anthocyanin biosynthetic genes.

### Hierarchical regulation of the MBW complex regulating anthocyanin biosynthesis

Several transcription factors have been reported to regulate anthocyanin biosynthesis via interaction with an activating MYB or MBW complex. For example, *AtLBD37*, *AtLBD38* and *AtLBD39* are negative regulators of anthocyanin biosynthesis via regulating *PAP1* and *PAP2* in *Arabidopsis thaliana* (Rubin *et al.*, 2009). *VmTDR4*, a MADS box transcription factor, promotes anthocyanin accumulation through direct or indirect control of the R2R3 MYB family in bilberry (Jaakola *et al.*, 2010). In apple, MdHY5 promotes anthocyanin accumulation by directly binding to the *MdMYB10* promoter (An *et al.*, 2017). In blood‐fleshed peach, BL, a NAC transcription factor, forms a heterodimer with PpNAC1 and activate the transcription of *PpMYB10.1* (Zhou *et al.*, 2015). In addition, some TFs play roles in anthocyanin accumulation via competing with the MYB or bHLH to interrupt or stabilize the MBW complex, such as AtMYBL2 and AtSPL9 (Dubos *et al.*, 2008; Gou *et al.*, 2011; Matsui *et al.*, 2008). Another anthocyanin activator AtTCP3, which interacts separately with PAP1, PAP2 and TT2, promotes anthocyanin accumulation via stabilizing the formation of the MBW complex and thus stimulating the expression of LBGs (Li and Zachgo, 2013). Further research has characterized TFs in the MYB, LBD, SPL, TCP, MADS, bZIP and NAC families affecting anthocyanin levels. Here, we characterized FaRAV1 involvement in anthocyanin biosynthesis via upstream activation of *FaMYB10*. Unlike MdERF1B, FaRAV1 activates *FaMYB10* transcription, but does not form a protein complex with FaMYB10 (Figure S7). FaERF85, the homologue of MdERF1B, might regulate anthocyanin via protein–DNA and protein–protein interaction, which needs further validation.

### *FaRAV1* positively regulates anthocyanin accumulation at multiple levels

As mentioned above, *FaRAV1* promotes anthocyanin accumulation by activating the *FaMYB10* promoter (Figure 1). Moreover, further investigation by luciferase transactivation assays, showed that FaRAV1 could also directly activate the promoters of anthocyanin biosynthetic structural genes. FaRAV1 showed the highest activation effect towards the *DFR* promoter compared with the other promoters (Figure 6a). Transcripts of *FaMYB10* and anthocyanin biosynthesis‐related genes were altered in transient OE and RNAi fruit accordingly (Figures 4 and 5). We also demonstrated that FaRAV1 can directly bind to the *CHS*, *F3H*, *DFR* and *GT1* promoters by yeast one‐hybrid assays (Figure 6b). However, FaRAV1 would not directly bind to the promoters of *CHI* and *ANS* (Figure 6b), indicating the activation of *CHI* and *ANS* is not directly driven by FaRAV1 and other TFs may be involved (e.g. FaMYB10). We also carried out *cis*‐element analysis of these anthocyanin biosynthesis promoters (Figure S8). CAACA motif  existed in all promoters of anthocyanin biosynthetic genes, which would not explain why FaRAV1 could not bind to the promoters of *CHI* and *ANS,* implying other AP2/ERFs might also be involved. Interestingly, another differentially expressed anthocyanin gene *RAP* (reduced anthocyanin in petioles) is significantly up‐regulated 8.1‐fold in OE fruit and down‐regulated 0.57‐fold in RNAi fruit (Figure S9). *RAP* encodes a glutathione S‐transferase (GST) gene, which is the principal transporter of anthocyanins and whose transcript is up‐regulated in *FvMYB10* OE fruit (Lin‐Wang *et al.*, 2014) and down‐regulated in Yellow Wonder fruit, a natural mutant of the *MYB10* gene (Gao *et al.*, 2019; Hawkins *et al.*, 2016; Luo *et al.*, 2018), suggesting *RAP* operates downstream of *FaMYB10*. Dual‐luciferase experiments showed that the promoter activity of *RAP* cannot be triggered by FaRAV1 (Figure S10), suggesting FaRAV1 regulates its transactivation via *FaMYB10.* CRISPR/Cas9 has become a tool for studying the gene function in plant and is now available in cultivated strawberry (Gao *et al.*, 2019). The use of CRISPR to generate new mutants will be one important feature of future work.

## Conclusions

**Figure 7:**
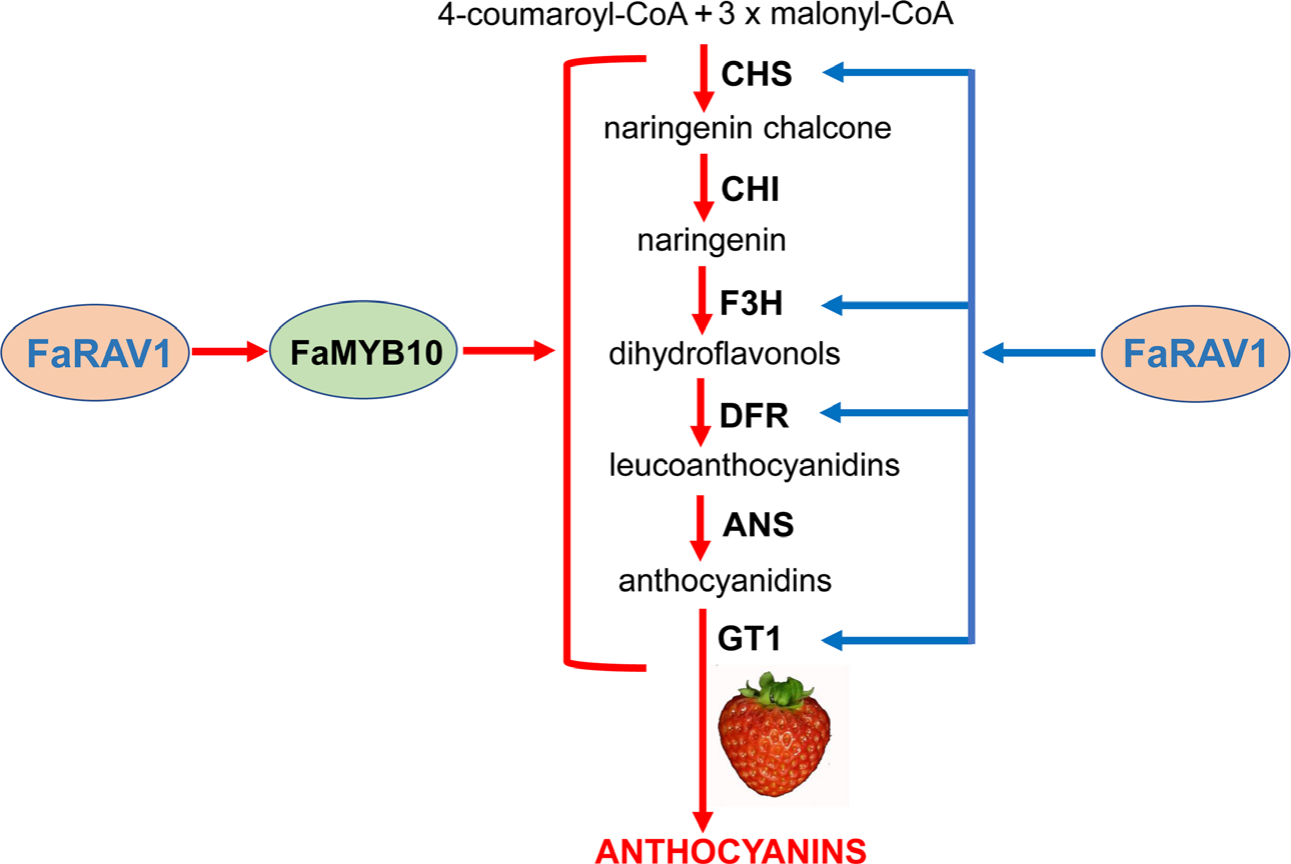
A working model of the role of FaRAV1 in promoting anthocyanin accumulation in strawberry fruit. FaRAV1 directly binds to the promoter of *FaMYB10* and activates its transcription (approximately 4.0‐fold), marked by red arrows. Blue arrows indicate FaRAV1 can directly bind to and activate *CHS* (1.53‐fold), *F3H* (1.95‐fold)*, DFR* (3.6‐fold) and *GT1* (2.3‐fold) promoters.

Despite the RAV gene family being characterized in many different physiological pathways in plants, the role of RAVs in anthocyanin biosynthesis has not been previously studied. Here, we found that FaRAV1 directly bound to and activated the promoter of *FaMYB10*. In addition, FaRAV1 can also directly bind to and activate *CHS*, *F3H*, *DFR* and *GT1* promoters, showing a second effect of *FaRAV1* on anthocyanin biosynthesis (Figure 7). Transient overexpression of *FaRAV1* in strawberry fruit increased anthocyanin‐related gene expression and promoted anthocyanin production. Correspondingly, transient RNAi of *FaRAV1* fruit contained less anthocyanin and lower level of anthocyanin‐related gene expression compared to control fruit. These results demonstrate that *FaRAV1* functions positively in strawberry anthocyanin accumulation.

## Experimental procedures

### Plant material and ABA treatment

Octoploid strawberry (*Fragaria* × *ananassa* ‘Yuexin’) plants were grown at the Zhejiang Academy of Agricultural Sciences in Haining (Zhejiang, China). Four stages of fruit (G, green; T, turning; IR, intermediate red; R, full red) were harvested at 25, 28, 33 and 37 days after anthesis and then transported to the laboratory within 2 h. Fruit of uniform size, absence of disease and mechanical wounding were selected. After removing the calyces, fruit were then separated into apical, middle and basal parts and frozen in liquid nitrogen rapidly and then stored at −80 °C for further use. Gene expression analyses are conducted with three biological replicates (representing ten fruit) for each time point. Green fruit were harvested for ABA treatment. Discs (10 mm in diameter and 2 mm in thickness) of fruit were prepared and infiltrated for 30 min in equilibration buffer (Archbold, 1999; Han *et al.*, 2015), after which different concentrations of ABA (Sigma‐Aldrich; A1049, Germany) were added and then shaken for 1 or 6 h at 25 °C. Equilibration buffer consisted of 50 mm MES‐Tris (pH = 5.5), 10 mm MgCl_2_, 10 mm EDTA, 5 mm CaCl_2_, 200 mm mannitol and 5 mm vitamin C. After incubation, the residues were dried with tissue and frozen rapidly in liquid nitrogen and kept at −80 °C until further use.

### RNA isolation and RT‐qPCR

Total RNA from strawberry fruit was extracted using the CTAB method (Chang *et al.*, 1993). After elimination of genome DNA by gDNA eraser, 1 µg RNA was used for first‐strand cDNA synthesis by the PrimeScript™ RT reagent Kit (Takara, Dalian, China) and then diluted with water (1: 20). Real‐time PCR was carried out using a CFX96 instrument with SsoFast EvaGreen Supermix Kit (Bio‐Rad, America). The specificity of primers was assured by both melting curves and product sequencing before use. The PCR reactions and mixture were as described in our previous report (Yin *et al.*, 2012). Data were analysed and relative expression level of the genes was calculated using the 2^(‐△△^ *^C^* ^t)^ method and using expression of the strawberry *FaRIB413* (Zorrilla‐Fontanesi *et al.*, 2012) as the internal control. The primers for RT‐qPCR analysis are listed in Table S1.

### Gene isolation, promoter cloning and analysis

The SK vectors of *FaAP2/ERFs* were described in our previous report (Zhang *et al.*, 2018b). Promoters of anthocyanin‐related genes were isolated according to genome databases (https://bioinformatics.psb.ugent.be/plaza/ or http://strawberry‐garden.kazusa.or.jp/index.html). The primers are listed in Table S2. These promoters were isolated and sequenced, and we performed an in silico analysis for *FaMYB10* (1061 bp) (Delgado *et al.*, 2018), *CHS* (1614 bp), *CHI* (2211 bp), *F3H* (1019 bp), *DFR* (1994 bp), *ANS* (830 bp), *GT1* (1526 bp) and *RAP* (1427 bp) from upstream of the start codon. The analysis of *cis*‐elements within *FaRAV1* promoter regions was conducted using the website http://bioinformatics.psb.ugent.be/webtools/plantcare/html/.

### Dual‐luciferase assay

Dual‐luciferase assay was applied to investigate the transactivation activities of different TFs on target promoters. The full‐length sequences of *FaAP2/ERFs* transcription factors were amplified and inserted into pGreen II 0029 62‐SK vector and the promoters of eight anthocyanin‐related genes were constructed in the pGreen II 0800‐LUC vector. The primers used for vector construction are listed in Table S2. All constructs were electroporated into *Agrobacterium tumefaciens* GV3101, and the cultures were adjusted to an OD_600_ of 0.75 with infiltration buffer (10 mm MES, 10 mm MgCl_2_, 150 mm acetosyringone, pH 5.6). To research the activity of a specific transcription factor towards the target promoter, a mixture of *A. tumefaciens* containing TFs (1 mL) and promoters (100 μL) was infiltrated into tobacco (*Nicotiana benthamiana*) leaves by needleless syringe. Tobacco plants were grown in a greenhouse with a light/dark cycle of 16: 8 h at 24 °C. Three days after infiltration, discs from the tobacco leaves were collected and enzyme activity of firefly and renilla luciferases was measured using dual‐luciferase reagents (Promega, America). For every TF–promoter interaction, three biological replicates were performed for individual experiment.

### Yeast one‐hybrid assay

The Matchmaker™ Gold Yeast One‐Hybrid Library Screening System (Clontech, Japan) was used to test the interaction of FaRAV1 with the *FaMYB10* promoter. The promoter sequence was amplified and inserted into pAbAi vector, and the full‐length *FaRAV1* was cloned into pGADT7 vector. The recombinant *FaMYB10* promoter–pAbAi vector was linearized and transformed into the Y1HGold yeast strain to test the promoter autoactivation according to the system user manual. The Y1HGold strain carrying *FaMYB10* promoter was transfected with the FaRAV1‐pGADT7 plasmid and the empty vector pGADT7 as a negative control separately.

### Recombinant protein and EMSA analysis

The full‐length *FaRAV1* was inserted into pET‐32a (Clontech, Japan) to generate the recombinant N‐terminal FaRAV1‐His fusion protein. The construct was purified and transformed into *Escherichia coli* strain Rosetta 2(DE3)pLysS (Novagen, Germany). The transformed cells were induced by 0.5 mm isopropyl *β*‐D‐1‐thiogalactopyranoside (IPTG) followed by incubation at 16 °C for 20 h. Then, the cells were collected by centrifugation and resuspended in buffer (20 mm Tris‐HCl, pH = 8.0, 0.5 m NaCl), after which they were subjected to sonication on ice with 2‐s/4‐s on/off cycle for 20 min, centrifuged at 10000 rpm for 30 min at 4 °C and the supernatant was purified using a HisTALON™ Gravity Column (Clontech, Japan), following the steps described in the official user manual.

EMSA was performed by using the LightShift Chemiluminescent EMSA kit (Thermo, America ) according to the manufacturer’s instructions. Single‐strand oligonucleotides were synthesized and 3′‐biotin‐end‐labelled by HuaGene. The details of the EMSA experiment can be found in Ge *et al. *(2017). The EMSA probes are listed in Table S2.

### Anthocyanin measurement

Strawberry fruit were ground to powder under liquid nitrogen. Approximately 1 g powder was added to 5 mL methanol‐0.05% HCl and then extracted at 4 °C in the dark for 12 h. The supernatant was collected by centrifugation for further analysis and the extraction procedure was repeated once (Wrolstad *et al.*, 1982). The collected supernatants were pooled, filtered through 0.22 µm Millipore membranes and then evaporated at 30 °C in an evaporator machine. The residual material was resuspended in 1 mL methanol and filtered through a 0.22‐µm Millipore membrane for HPLC analysis using an HPLC (Agilent 1269, America) analytical column SB‐C18 (4.6 × 250 mm, 5 µm, Agilent Technologies, America).

The detection procedure was set as solvent A (formic acid: water, 1: 1000, v/ v) and solvent B (formic acid: acetonitrile, 1: 1000, v/ v) with the following gradient: 0–2 min, 5%; 2–7 min, 5–15%; 7–20 min, 15–20%; 20–25 min, 20–27%; 25–32 min, 27%; 31–41 min, 27%–35%; 41.01–43 min, 5%. The flow rate was 0.8 mL/min at 30 °C. The post‐run‐time was set at 5 min and the detection wavelength was 520 nm (Cheng *et al.*, 2014). Pelargonidin‐3‐glucoside (P3G) and cyanidin‐3‐glucoside (C3G) were used as standards.

### Phylogenetic tree construction

The phylogenetic tree was constructed with the FigTree v1.4.2 program, aligning the full‐length amino acid sequences of RAVs using the neighbour‐joining method for the ClustalX v2.0 program. The sequences using for the phylogenetic tree included *Fragaria* × *ananassa* FaRAV1, FaRAV2, FaRAV3, FaRAV4, FaRAV5, FaRAV6, *Arabidopsis thaliana* AtRAV1 (At1g13260), AtRAV1‐like (At3g25730), AtTEM1 (At1g25560), AtTEM2 (At1g68840), AtRAV3 (At1g50680), AtRAV3L (At1g51120), *Solanum lycopersicum* SlRAV1, SlRAV2, SlRAV3, *Malus* × *domestica* MdRAV1 (MDP0000939633), MdRAV2 (MDP0000128924), MDP0000945267, MDP0000321569, MDP0000223137, MDP0000153589, MDP0000165802, MDP0000534780, MDP0000526584, MDP0000485280, MDP0000207722, *Oryza sativa* OsRAV1 (Os01g04800.1), OsRAV2 (Os01g04750.1), OsRAV3 (Os05g47650.1), OsRAV4 (Os01g49830.1), *Populus trichocarpa* PtRAV1, PtRAV2 (GenBank_Number), PtRAV3, PtRAV4 and PtRAV5 .

### Subcellular localization analysis

The *FaRAV1* full‐length coding sequence without the stop codon was fused to the pCAMBIA1300‐sGFP vector (*KpnI*/*SalI*) at the C‐terminal and then expressed transiently in transgenic *N. benthamiana* (with nucleus‐located mCherry) leaves by *A. tumefaciens* infiltration (GV3101) using the same method as described above for the dual‐luciferase assay. Tobacco leaves were measured 2 days after infiltration and the fluorescence was imaged with a Nikon A1‐SHS confocal laser scanning microscope. The excitation wavelength for GFP fluorescence was 488 nm, and fluorescence was detected at 490 to 520 nm. The primers for GFP construction are listed in Table S2.

### Transient overexpression and RNAi in strawberry fruit

The construct of pGreenII 0029 62‐SK containing *FaRAV1* was used for transient overexpression. Forward and reverse PCR‐amplified cDNA fragments of *FaRAV1* were inserted into the 2× CaMV35S‐driven vector pHB to produce the *FaRAV1*‐RNAi construct. All the constructs were independently transformed into *A. tumefaciens* strain GV3101. Attached fruit of similar size at the green (G) stage were selected and injected with *A. tumefaciens*, containing construct *FaRAV1*‐SK and empty vector SK, *FaRAV1*‐RNAi and empty vector pHB under the same infiltration conditions, which were performed in 2018 and 2019 respectively. The cultures were adjusted to an OD_600_ of 1.0 with infiltration buffer (10 mm MES, 10 mm MgCl_2_, 150 mm acetosyringone, pH 5.6). *A. tumefaciens* suspension was evenly injected into the basal part of fruit at two or three sites until the whole fruit became hydrophanous. The fruit were collected 9 days after transfection and each fruit was collected as an individual sample. Three biological replicates were sampled for analysis. The primers for RNAi construction are listed in Table S2.

### RNA‐seq

The *FaRAV*1 transient overexpressing fruit and relevant control fruit were processed for Illumina RNA‐seq analysis. Cultivated strawberry genome‐based reads used a reference for transcriptome analysis. TPM (Transcripts per million reads) were used to estimate gene expression levels and a threshold of twofold change was applied to select differentially expressed genes.

### Yeast two‐hybrid assay

Yeast two‐hybrid assay was performed to test the interaction between FaRAV1 and FaMYB10 using the Matchmaker™ Gold Yeast Two‐Hybrid System (Clontech, Japan). Full‐length coding sequences of *FaRAV1* and *FaMYB10* were separately cloned into pGADT7 and pGBKT7 vectors. The concentrations of aureobasidin A (AbA) to inhibit self‐transactivation were tested on SD/‐Trp medium. pGBKT7‐p53 and pGBKT7‐T were used as positive control, while pGBKT7‐Lam and pGBKT7‐T were used as negative control. pGBKT7 and pGADT7 vectors containing target genes were co‐transformed into the Y2H strain and the interactions were detected on QDO (SD/‐Ade/‐His/‐Leu/‐Trp) in the presence of AbA and X‐α‐Gal. The primers for vector construction are listed in Table S2.

### Statistics

Student’s two‐tailed *t*‐test (*, *P* < 0.05; **, *P* < 0.01; ***, *P* < 0.001) was used to evaluate significant differences between two groups in this study. Figures were treated with Origin 8.0 (Microcal Software, America). Least significant differences (LSD) at the 5% level were conducted by DPS7.05 (Zhejiang University).

### Accession numbers

GenBank accession number for the genes identified are *FaRAV1*, *XM_011466945.1*; *FaMYB10*, EU155162; *CHS*, AY997297; *CHI*, AB201755; *F3H,* AY691919; *DFR*, AY695812; *ANS,* AY695817; and *GT1,* AY575056.

## Conflict of interest

The authors declare no conflict of interest.

## Author contributions

K.C. and G.J. conceived the research plans; Y.S., X.r.Y. and K.C. supervised the experiments; Z.Z., Y.M., Y.Z., Y.X., W.L. and Y.L. performed the experiments and analysis; S.L., X.L. and X.f.Y. provided technical assistance to Z.Z.; Z.Z. and Y.S. wrote the article. D.G. and A.C.A. were involved in the design, discussion and revision of the manuscript; all authors read and approved the final article.

## Supporting information


**Figure S1** Phylogenetic tree analysis of strawberry AP2/ERF proteins and related AP2/ERF TFs from apple and pear.
**Figure S2** Phylogenetic analysis of FaRAV1 and 34 other RAV proteins.
**Figure S3** Subcellular localization of FaRAV1. *FaRAV1* was inserted into the pCAMBIA1300‐sGFP vector and transiently expressed in tobacco leaves.
**Figure S4** The appearance of *FaRAV1* overexpression fruit (right) 9 d after injection, compared to the control (left).
**Figure S5** PEGG analysis of DEGs between *FaRAV1* OE fruit and control fruit.
**Figure S6** RT‐qPCR analysis of *FaRAV1* expression in response to ABA treatment in strawberry fruit.
**Figure S7** Yeast two‐hybrid analysis of the interactions between FaRAV1 and FaMYB10.
**Figure S8** *Cis* regulatory elements in promoters of anthocyanin biosynthetic genes.
**Figure S9** Relative expression of *RAP* in *FaRAV1* OE and RNAi fruit compared with the control fruit.
**Figure S10** Regulatory effect of FaRAV1 on the promoter of *RAP*.
**Table S1** Primers used for reverse transcription quantitative PCR.
**Table S2** Primers used for vector construction.
**Table S3** Motifs in the *FaRAV1* promoter identified in silico by PlantCARE.
