# Supplementary material for: The strawberry transcription factor FaRAV1 positively regulates anthocyanin accumulation by activation of FaMYB10 and anthocyanin pathway genes
Source: Plant Biotechnol J. 2020 Apr 13;18(11):2267–79. doi: 10.1111/pbi.13382 (PMC7589338; doi:10.1111/pbi.13382)
Supplement: Supplementary file 1 — Figure S1 Phylogenetic tree analysis of strawberry AP2/ERF proteins and related AP2/ERF TFs from apple and pear. Figure S2 Phylogenetic analysis of FaRAV1 and 34 other RAV proteins. Figure S3 Subcellular localization of FaRAV1. FaRAV1 was inserted into the pCAMBIA1300‐sGFP vector and transiently expressed in tobacco leaves. Figure S4 The appearance of FaRAV1 overexpression fruit (right) 9 d after injection, compared to the control (left). Figure S5 PEGG analysis of DEGs between FaRAV1 OE fruit and control fruit. Figure S6 RT‐qPCR analysis of FaRAV1 expression in response to ABA treatment in strawberry fruit. Figure S7 Yeast two‐hybrid analysis of the interactions between FaRAV1 and FaMYB10. Figure S8 Cis regulatory elements in promoters of anthocyanin biosynthetic genes. Figure S9 Relative expression of RAP in FaRAV1 OE and RNAi fruit compared with the control fruit. Figure S10 Regulatory effect of FaRAV1 on the promoter of RAP. Table S1 Primers used for reverse transcription quantitative PCR. Table S2 Primers used for vector construction. Table S3 Motifs in the FaRAV1 promoter identified in silico by PlantCARE. [file PBI-18-2267-s001.pdf]

FaRAV1 positively regulates anthocyanin accumulation

Supplemental Data

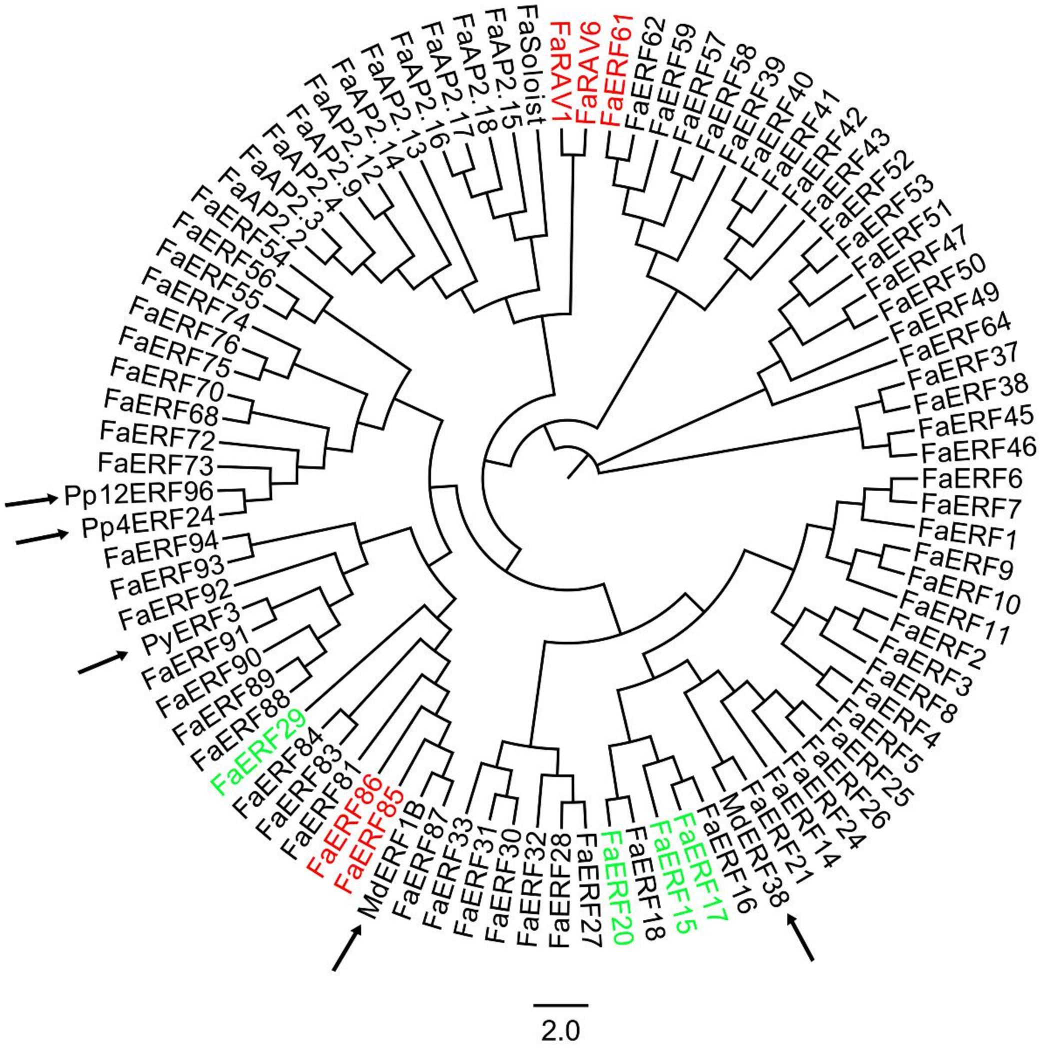

Supplemental Figure S1. Phylogenetic tree analysis of strawberry AP2/ERF proteins and related AP2/ERF TFs from apple and pear. Those ERFs that activated *FaMYB10* promoter are denoted in red, which repressed *FaMYB10* promoter are denoted in green. Arrows indicted that the five ERFs which have been characterized in anthocyanin biosynthesis in apple and pear. NCBI accession numbers of related AP2/ERF TFs in apple and pear are as follows: MdERF1B (XM_008342898.2), MdERF38 (XM_029104540.1), PyERF3 (MF489220), Pp4ERF24 (XM_009335420.2) and Pp12ERF96 (XM_009370895.2).

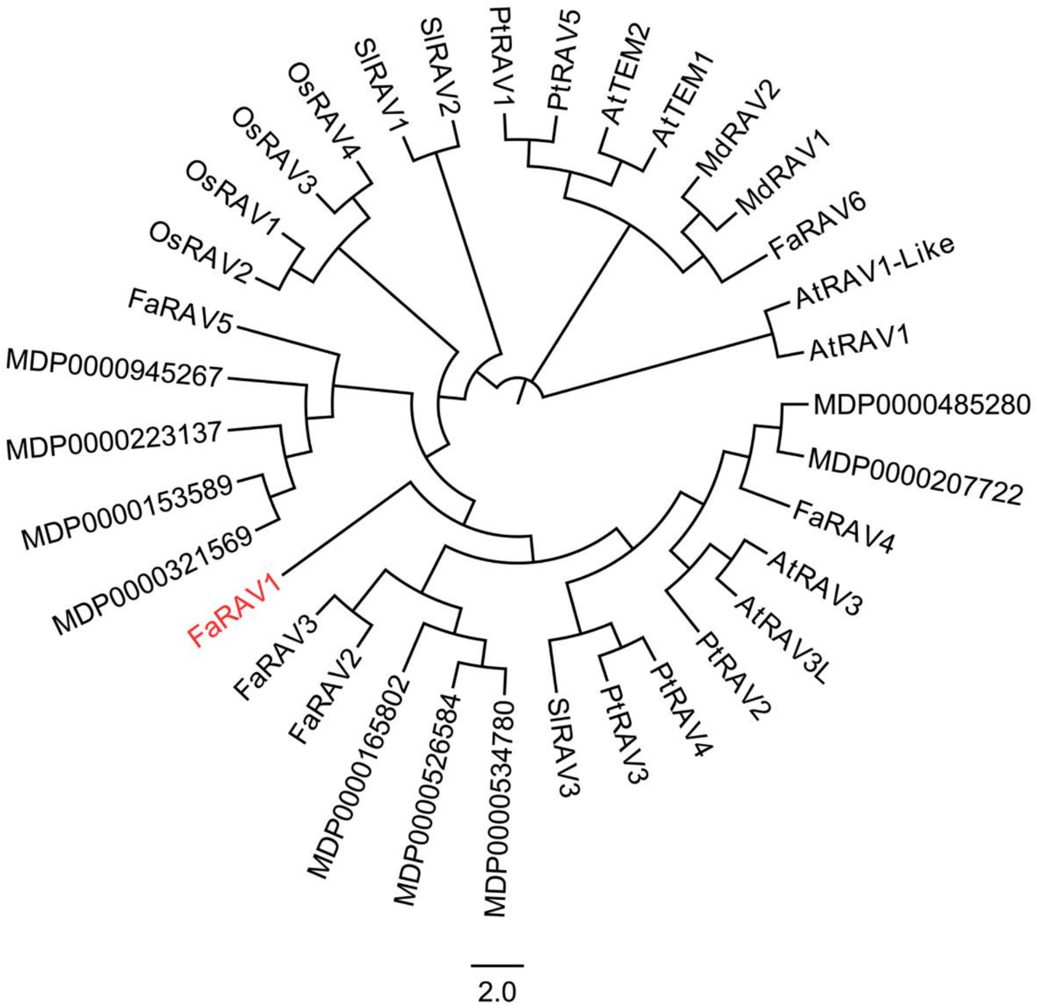

Supplemental Figure S2. Phylogenetic analysis of FaRAV1 and 34 other RAV proteins. All the sequences were obtained from the NCBI database. FaRAV1 is denoted in red. NCBI accession numbers: *Fragaria* × *ananassa* FaRAV1 (XM_011466945.1), FaRAV2 (XM_004309888.2), FaRAV3 (XM_004301388.2), FaRAV4 (XM_004296255.2), FaRAV5 (XM_011469524.1), FaRAV6 (XM_004297092.2), *Arabidopsis thaliana* AtRAV1 (At1g13260), AtRAV1-like (At3g25730), AtTEM1 (At1g25560), AtTEM2 (At1g68840), AtRAV3 (At1g50680), AtRAV3L (At1g51120), *Solanum lycopersicum* SlRAV1 (XM_004236951.3), SlRAV2 (XM_001320461.1), SlRAV3 (XM_010319814.1), *Malus* × *domestica* MdRAV1 (MDP0000939633), MdRAV2 (MDP0000128924), MDP0000945267, MDP0000321569, MDP0000223137, MDP0000153589, MDP0000165802, MDP0000534780, MDP0000526584, MDP0000485280, MDP0000207722, *Oryza sativa* OsRAV1 (Os01g04800.1), OsRAV2 (Os01g04750.1), OsRAV3

(Os05g47650.1), OsRAV4 (Os01g49830.1), *Populus trichocarpa* PtRAV1 (XP_002315958.2), PtRAV2 (XP_002304025.1), PtRAV3 (XP_024446356.1), PtRAV4 (XP_024458800.1), PtRAV5 (XP_002311438.2).

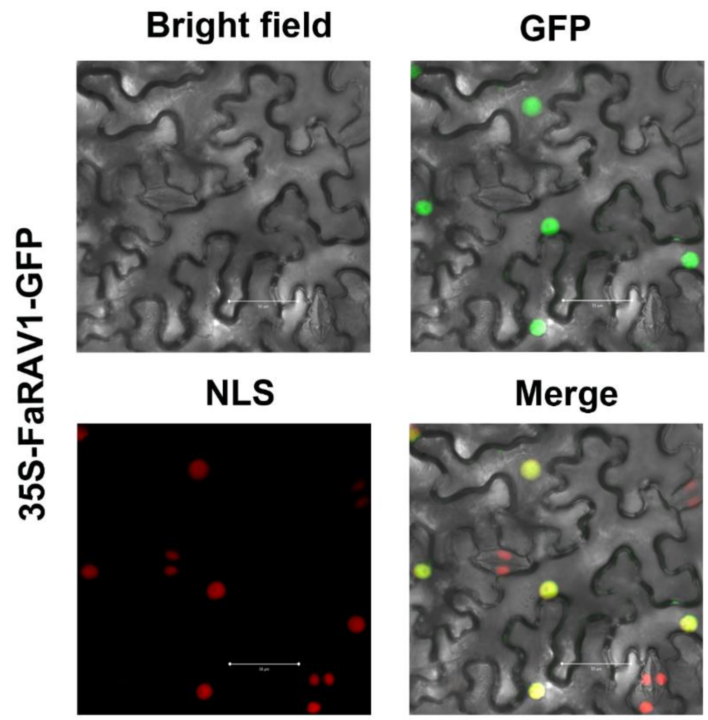

Supplemental Figure S3. Subcellular localization of FaRAV1. *FaRAV1* was inserted into the pCAMBIA1300-sGFP vector and transiently expressed in tobacco leaves. The GFP fluorescence indicated the localization of *FaRAV1*. The tobacco was stably transformed with a specific nucleus-localized red fluorescent protein, mCherry and the red fluorescence indicated the nucleus-localized signal (NLS). Bars = 50 μm.

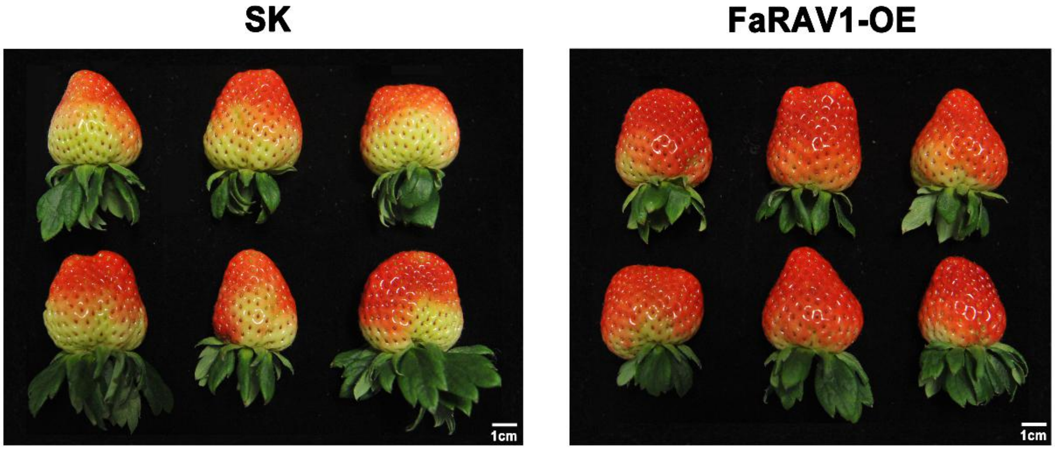

Supplemental Figure S4. The appearance of *FaRAV1* overexpression fruit (right) 9 d after injection, compared to the control (left).

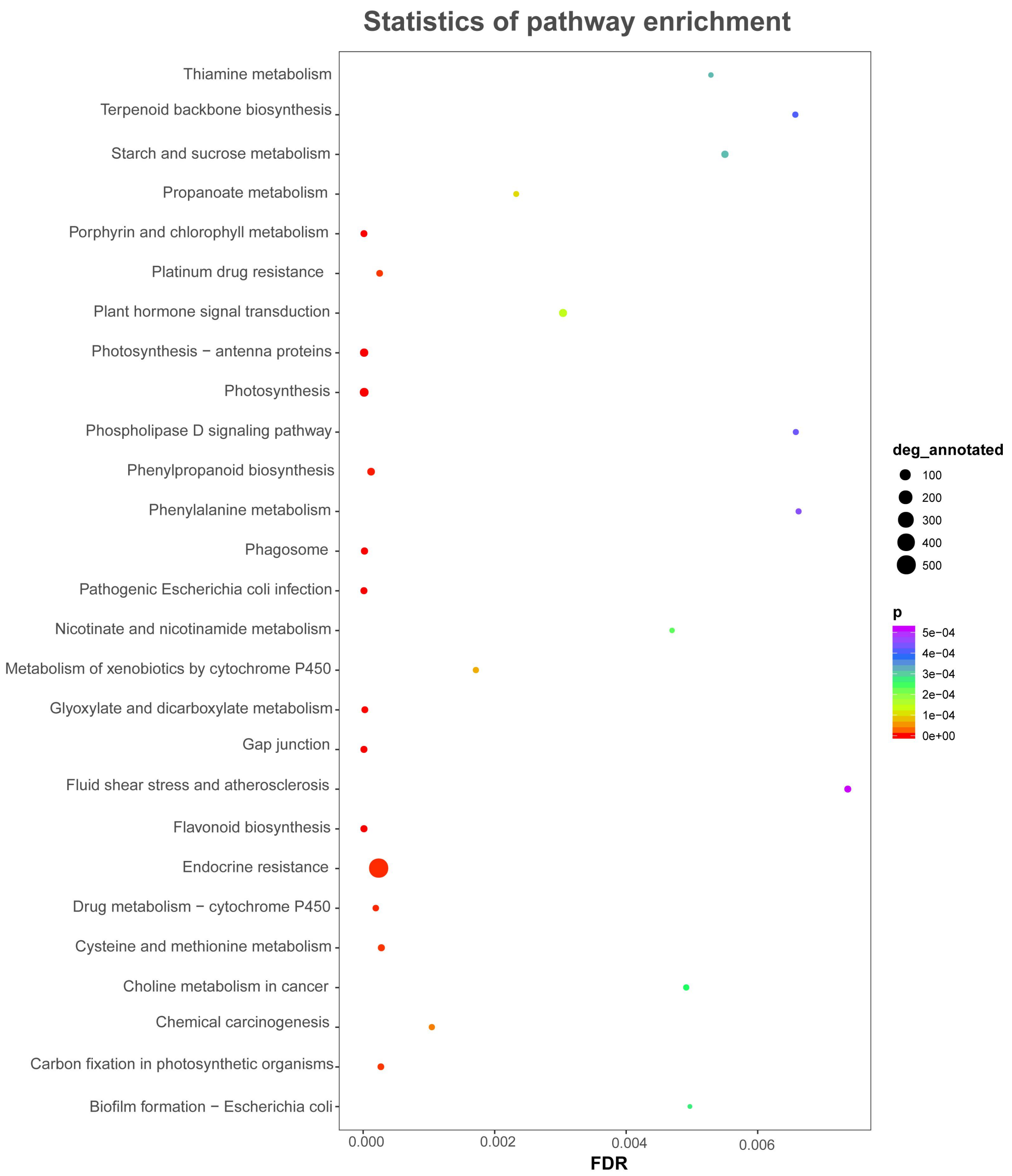

Figure S5. PEGG analysis of DEGs between *FaRAV1* OE fruit and control fruit.

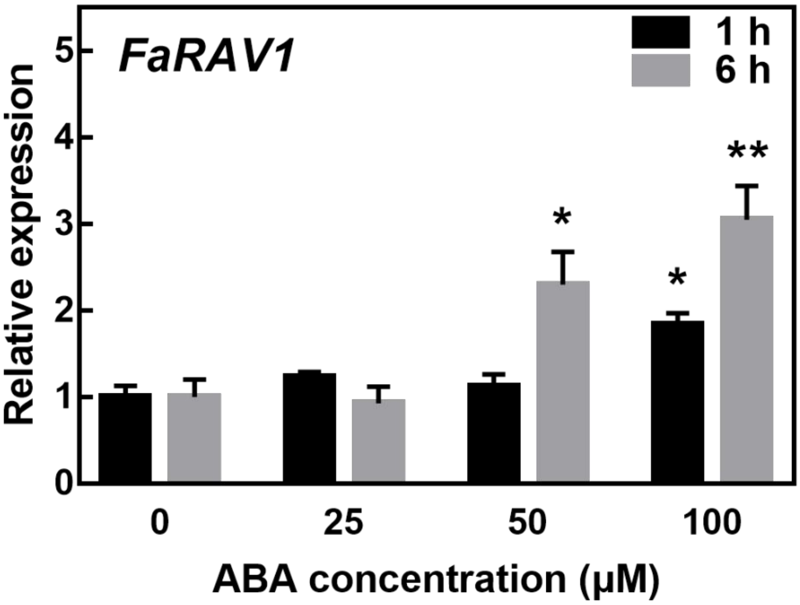

Supplemental Figure S6. RT-qPCR analysis of *FaRAV1* expression in response to ABA treatment in strawberry fruit. Detached fruit slices at the green stage were treated with different concentrations of ABA for 1 or 6 h and then *FaRAV1* expression was tested. Error bars are the SE of three biological replicates. Asterisks denote significant differences using Student's t test, *$P<0.05$, **$P<0.01$.

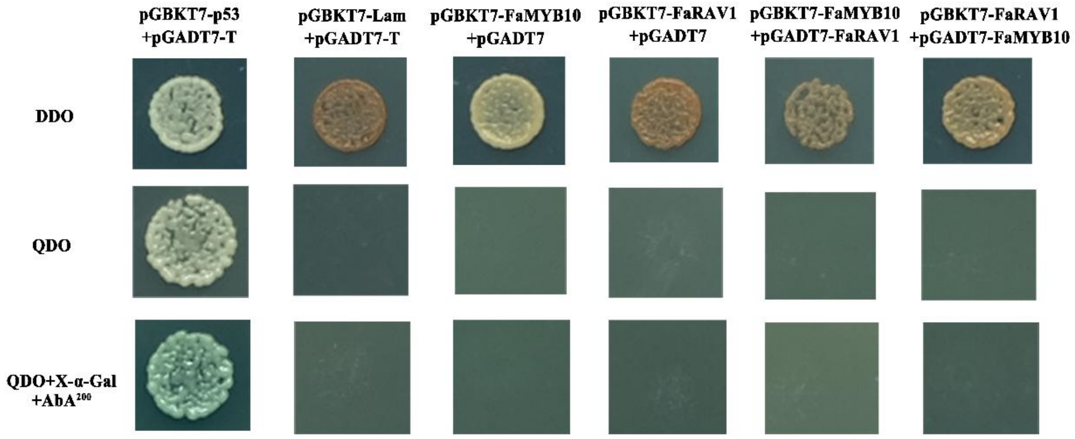

Supplemental Figure S7. Yeast two-hybrid analysis of the interactions between FaRAV1 and FaMYB10. FaRAV1 and FaMYB10 were separately fused into pGADT7 and pGBKT7 vectors. pGBKT7-p53 and pGBKT7-T were used as positive control, while pGBKT7-Lam and pGBKT7-T were used as negative control. Protein-protein interactions were detected on SD medium lacking Leu, Trp, His and Ade, with AbA^{200} and X-α-Gal.

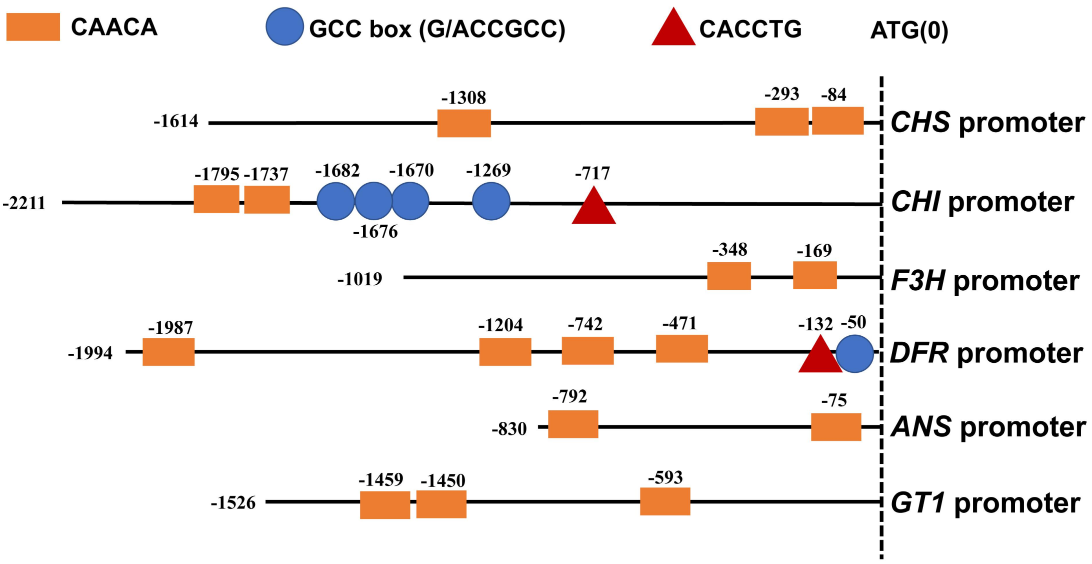

Supplemental Figure S8. *Cis* regulatory elements in promoters of anthocyanin biosynthetic genes. Putative AP2/ERF binding sites: CAACA motif, GCC box (A/GCCGCC), CACCTG motif are indicated in the promoter regions of genes encoding the anthocyanidin synthase.

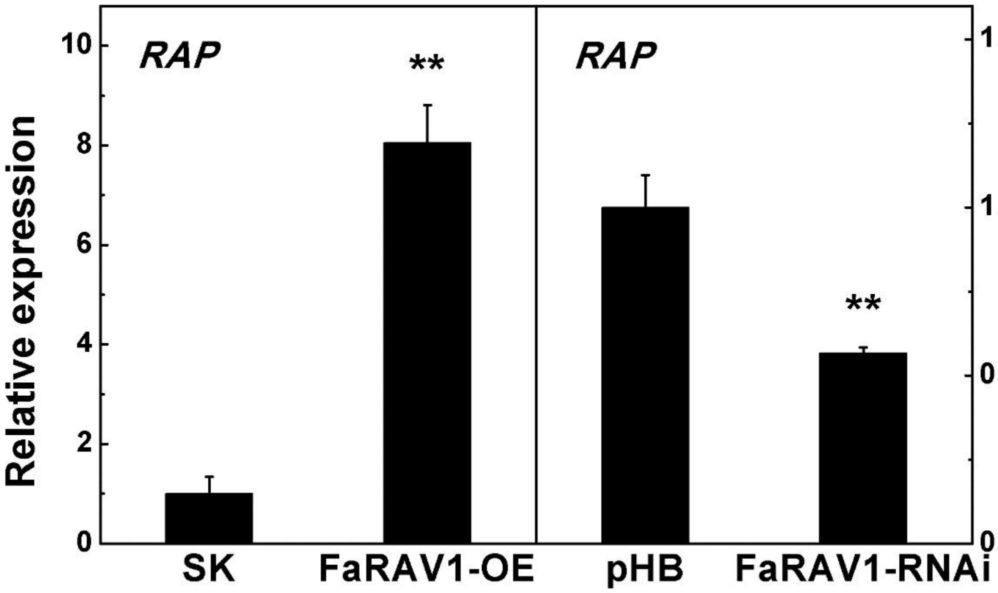

Supplemental Figure S9. Relative expression of *RAP* in *FaRAV1* OE and RNAi fruit compared with the control fruit. Asterisks denote significant differences using Student's t test, $^{**}P<0.01$.

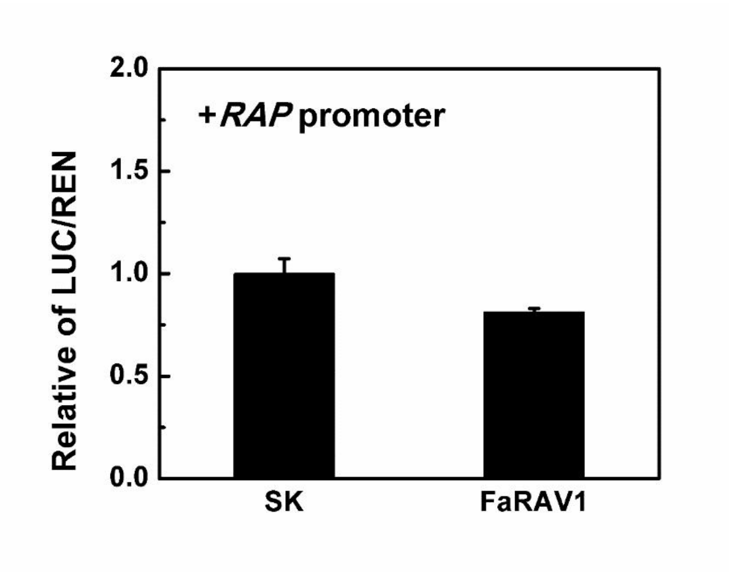

Supplemental Figure S10. Regulatory effect of FaRAV1 on the promoter of *RAP*.

Supplemental Table S1. Primers used for reverse transcription quantitative PCR.

**Gene**	**Forward (5'-3')**	**Reverse (5'-3')**
FaRIB413	ACCGTTGATTCGCACAATTGGTCATCG	TACTGCGGGTCGGCAATCGGACG
FaRAV1	AGAGCGCATTCCAAAGTCAG	CACAATCCCCTGTTCCTTCA
FaMYB10	AGACGGCTTCATACGCAAAGA	ATGAAGGTTCGTGGTCGAGG
CHS	GGCTCACCGTCGAGACCG	GGAGAAGATCACTCGAATCA
CHI	GCCGGAAATGGGAAAGTG	GCTCAGTTTCATGCCTTGAC
F3H	CCCTAAGGTGGCCTACAACCAAT	CTTCTTGCAAATCTCAGCGC
DFR	CACGATTCACGACATTGCGAAATT	GAACTCAAACCCCATCTCTTTCAGCTT
ANS	GAAGTGCGTACCCAACTCCATCGT	ACCTTCTCCTTGTTGACGAGCCC
GT1	CCTGGCGCATGGTTCAGT	CGAGTTCCAACCGCAATGT
RAP	GCTACTACTGCTCGATCACC	TGAAGAGACTCGCAAAAGACA
FaRAV1-RNAi	TGGGAGCGGAGATTACATTC	TGGTGAAATACGAAAGGCTGT

Supplemental Table S2. Primers used for vector construction.

**Primer name**	**Sequence (5’-3’)**
**LUC: `**	
FaMYB10pro-LUC-FP-BamHI	CAGCCCGGGGGATCCAAAAAAATGGTACAATTAAG
FaMYB10pro-LUC-RP-NcoI	TGGCGTCTTCCATGGGAAAATTAAGCAGATTTC
CHSpro-LUC-FP-BamHI	CAGCCCGGGGGATCCGAAAATTAAAATGGTGGGGG
CHSpro-LUC-RP-NcoI	TGGCGTCTTCCATGGTTTGATTTCTCAGAGAAGTG
CHIpro-LUC-FP-BamHI	CAGCCCGGGGGATCCCATTTTCCCGGAGAGATG
CHIpro-LUC-RP-NcoI	TGGCGTCTTCCATGGTTGATTTTCTTGGTTTTG
F3Hpro-LUC-FP-BamHI	CAGCCCGGGGGATCCGCAATGATTGCCTATAATTA
F3Hpro-LUC-RP-NcoI	TGGCGTCTTCCATGGTCTGATATATGCTCTCTAGC
DFRpro-LUC-FP-BamHI	CAGCCCGGGGGATCCTTGCCCCCAACAAAGGAATT
DFRpro-LUC-RP-NcoI	TGGCGTCTTCCATGGGCCTAGCTAGTTAGTGCCGT
ANSpro-LUC-FP-BamHI	CAGCCCGGGGGATCCAACTCCGATCCCGAACAAGT
ANSpro-LUC-RP-NcoI	TGGCGTCTTCCATGGTGTCGCTTCTAGCTATAGCT
GT1pro-LUC-FP-BamHI	CAGCCCGGGGGATCCCACATTCTTGTACTGAACTT
GT1pro-LUC-RP-NcoI	TGGCGTCTTCCATGGTTCTAGCTAGTCAAGCTACT
RAPpro-LUC-FP-BamHI	CAGCCCGGGGGATCCCAGGAGACTTCGTCGTCGGA
RAPpro-LUC-RP-NcoI	TGGCGTCTTCCATGGTATAGCCACCTGAAAACCTC
**pAbAi:**	
FaMYB10pro-pAbAi-FP-SacI	AAGCTTGAATTCGAGCTCAAAAAAATGGTACAATTAAG
FaMYB10pro-pAbAi-RP-XhoI	AGCACATGCCTCGAGGAAAATTAAGCAGATTTC
CHSpro-pAbAi-FP-SacI	AAGCTTGAATTCGAGCTCGAAAATTAAAATGGTGGGGG
CHSpro-pAbAi-RP-XhoI	AGCACATGCCTCGAGTTTGATTTCTCAGAGAAGTG
CHIpro-pAbAi-FP-SacI	AAGCTTGAATTCGAGCTCCATTTTCCCGGAGAGATG
CHIpro-pAbAi-RP-XhoI	AGCACATGCCTCGAGTTGATTTTCTTGGTTTTG
F3Hpro-pAbAi-FP-SacI	AAGCTTGAATTCGAGCTCGCAATGATTGCCTATAATTA
F3Hpro-pAbAi-RP-XhoI	AGCACATGCCTCGAGTCTGATATATGCTCTCTAGC
DFRpro-pAbAi-FP-SacI	AAGCTTGAATTCGAGCTCTTGCCCCCAACAAAGGAATT

DFRpro-pAbAi-RP-XhoI	AGCACATGCCTCGAGGCCTAGCTAGTTAGTGCCGT
ANSpro-pAbAi-FP-SacI	AAGCTTGAATTCGAGCTCAACTCCGATCCCGAACAAGT
ANSpro-pAbAi-RP-XhoI	AGCACATGCCTCGAGTGTCGCTTCTAGCTATAGCT
GT1pro-pAbAi-FP-SacI	AAGCTTGAATTCGAGCTCCACATTCTTGTACTGAACTT
GT1pro-pAbAi-RP-XhoI	AGCACATGCCTCGAGTTCTAGCTAGTCAAGCTACT
**GFP:**	
FaRAV1-GFP-FP-KpnI	GACGAGCTCGGTACCATGTCGATAGATCTCAGTTC
FaRAV1-GFP-RP-SalI	GCTCACCATGTCGACACCACTAATCTGAACACCAA
**pGADT7:**	
FaRAV1-AD-FP-EcoRI	GAGGCCAGTGAATTCATGTCGATAGATCTCAGTT
FaRAV1-AD-RP-BamHI	GAGCTCGATGGATCCTTAACCACTAATCTGAACAC
FaMYB10-AD-FP-EcoRI	GAGGCCAGTGAATTCATGGAGGGTTATTTCGGT
FaMYB10-AD-RP-BamHI	GAGCTCGATGGATCCTCATACGTAGGAGATGTTGA
**pGBKT7:**	
FaRAV1-BD-FP-EcoRI	ATGGAGGCCGAATTCATGTCGATAGATCTCAGTT
FaRAV1-BD-RP-BamHI	CAGGTCGACGGATCCTTAACCACTAATCTGAACAC
FaMYB10-BD-FP-EcoRI	ATGGAGGCCGAATTCATGGAGGGTTATTTCGGT
FaMYB10-BD-RP-BamHI	CAGGTCGACGGATCCTCATACGTAGGAGATGTTGA
**SK:**	
FaRAV1-SK-FP-BamHI	TAGAACTAGTGGATCCATGTCGATAGATCTCAGTT
FaRAV1-SK-RP-EcoRI	GCTTGATATCGAATTCTTAACCACTAATCTGAACAC
**PET:**	
FaRAV1-PET-FP-BamHI	GCTGATATCGGATCCATGTCGATAGATCTCAG
FaRAV1-PET-RP-NotI	TTGTCGACGGCGGCCGCACCACTAATCTGAACAC
**RNAi:**	
FaRAV1-FP1-pSK	GCTCTCGAGTCTAGAATGTCGATAGATCTCAGTTC
FaRAV1-RP1-pSK	CACAAGCTTGACACATTCATACACAATCC
FaRAV1-FP2-pSK	GGTGGATCCATGTCGATAGATCTCAGTTC
FaRAV1-RP2-pSK	CACGAATTCGACACATTCATACACAATCC

**EMSA:**	
Probe-FP-biotin	AGGGTGGGGACTCGACAGCAACAAGCTAACTTCCACATGCAATGGATCGAAAT
Probe-RP-biotin	ATTTCGATCCATTGCATGTGGAAGTTAGCTTGTTGCTGTCGAGTCCCCACCCT
Probe-FP	AGGGTGGGGACTCGACAGCAACAAGCTAACTTCCACATGCAATGGATCGAAAT
Probe-RP	ATTTCGATCCATTGCATGTGGAAGTTAGCTTGTTGCTGTCGAGTCCCCACCCT
Mutant-Probe-FP-biotin	AGGGTGGGGACTCGACAGTTTTTAGCTAACTTCCACATGCAATGGATCGAAAT
Mutant-Probe-RP-biotin	ATTTCGATCCATTGCATGTGGAAGTTAGCTAAAAACTGTCGAGTCCCCACCCT

Note: Added restriction enzyme sites are underlined.

Supplemental Table S3. Motifs in the *FaRAV1* promoter identified in silico by PlantCARE.

Elements	Sequences	Sites (+) = current strand; (-) = opposite strand	Putative functions
ABRE	ACGTG	1412 (-), 1243 (-)	*Cis*-acting element involved in the abscisic acid responsiveness
AE-box	AGAAACAA	1504 (+)	Part of a module for light response
ARE	AAACCA	1470 (+)	*Cis*-acting regulatory element essential for the anaerobic induction
AT-rich element	ATAGAAATCAA	589 (+)	Binding site of AT-rich DNA binding protein (ATBP-1)
AuxRR-core	GGTCCAT	425 (+)	*Cis*-acting regulatory element involved in auxin responsiveness
Box 4	ATTAAT	1216 (-)	Part of a conserved DNA module involved in light responsiveness
CAAT-box	CAAAT, CCAAT	949 (-), 1743 (+), 247 (+), 504 (+)	Common *cis*-acting element in promoter and enhancer regions
CAT-box	GCCACT	368 (-)	*Cis*-acting regulatory element related to meristem expression
CGTCA-motif	CGTCA	1007 (+)	*Cis*-acting regulatory element involved in the MeJA-responsiveness
G-box	CACGTC	1243 (+), 1412 (+)	*Cis*-acting regulatory element involved in light responsiveness
I-box	AAGATAAGGCT	1098 (-)	Part of a light responsive element
MRE	AACCTAA	1482 (-)	MYB binding site involved in light responsiveness
P-box	CCTTTTG	782 (+), 1519 (+)	Gibberellin-responsive element
TGACG-motif	TGACG	1007 (-)	*Cis*-acting regulatory element involved in the MeJA-responsiveness
